# Loss of non-coding RNA expression from the *DLK1-DIO3* imprinted locus correlates with reduced neural differentiation potential in human embryonic stem cell lines

**DOI:** 10.1186/scrt535

**Published:** 2015-01-05

**Authors:** Chu-Fan Mo, Fang-Chun Wu, Kang-Yu Tai, Wei-Chun Chang, Kai-Wei Chang, Hung-Chih Kuo, Hong-Nerng Ho, Hsin-Fu Chen, Shau-Ping Lin

**Affiliations:** Institute of Biotechnology, National Taiwan University, Taipei, 106 Taiwan; Department of Obstetrics & Gynecology, College of Medicine and the Hospital, National Taiwan University Hospital, Taipei, 100 Taiwan; Graduate Institute of Medical Genomics and Proteomics, College of Medicine, National Taiwan University, Taipei, 100 Taiwan; Genome and Systems Biology Degree Program, National Taiwan University, Taipei, 106 Taiwan; Genome and Systems Biology Degree Program, Academia Sinica, Taipei, 115 Taiwan; Genomic Research Center, Academia Sinica, Taipei, 115 Taiwan; Institute of Cellular and Organismic Biology, Academia Sinica, Taipei, 115 Taiwan; Agricultural Biotechnology Research Centre, Academia Sinica, Taipei, 115 Taiwan; Research Centre for Developmental Biology and Regenerative Medicine, National Taiwan University, Taipei, 106 Taiwan; Centre for Systems Biology, National Taiwan University, Taipei, 106 Taiwan

## Abstract

**Introduction:**

Pluripotent stem cells are increasingly used to build therapeutic models, including the transplantation of neural progenitors derived from human embryonic stem cells (hESCs). Recently, long non-coding RNAs (lncRNAs), including delta-like homolog 1 gene and the type III iodothyronine deiodinase gene (*DLK1-DIO3*) imprinted locus-derived maternally expressed gene 3 (*MEG3*), were found to be expressed during neural development. The deregulation of these lncRNAs is associated with various neurological diseases. The imprinted locus *DLK1-DIO3* encodes abundant non-coding RNAs (ncRNAs) that are regulated by differential methylation of the locus. We aim to study the correlation between the *DLK1-DIO3*-derived ncRNAs and the capacity of hESCs to differentiate into neural lineages.

**Methods:**

We classified hESC sublines into *MEG3*-ON and *MEG3*-OFF based on the expression levels of *MEG3* and its downstream microRNAs as detected by quantitative reverse transcription-polymerase chain reaction (qRT-PCR). A cDNA microarray was used to analyze the gene expression profiles of hESCs. To investigate the capacity of neural differentiation in *MEG3*-ON and *MEG3*-OFF hESCs, we performed neural lineage differentiation followed by neural lineage marker expression and neurite formation analyses via qRT-PCR and immunocytochemistry, respectively. *MEG3*-knockdown via small interfering RNA (siRNA) and small hairpin RNA (shRNA) was used to investigate the potential causative effect of *MEG3* in regulating neural lineage-related gene expression.

**Results:**

*DLK1-DIO3*-derived ncRNAs were repressed in *MEG3*-OFF hESCs compared with those in the *MEG3*-ON hESCs. The transcriptome profile indicated that many genes related to nervous system development and neural-type tumors were differentially expressed in *MEG3*-OFF hESCs. Three independent *MEG3*-knockdown assays using different siRNA and shRNA constructs consistently resulted in downregulation of some neural lineage genes. Lower expression levels of stage-specific neural lineage markers and reduced neurite formation were observed in neural lineage-like cells derived from *MEG3*-OFF-associated hESCs compared with those in the *MEG3*-ON groups at the same time points after differentiation.

**Conclusions:**

Repression of ncRNAs derived from the *DLK1-DIO3* imprinted locus is associated with reduced neural lineage differentiation potential in hESCs.

**Electronic supplementary material:**

The online version of this article (doi:10.1186/scrt535) contains supplementary material, which is available to authorized users.

## Introduction

Pluripotent stem cells are increasingly used for therapeutic models, including the transplantation of neural progenitors derived from human embryonic stem cells (hESCs) [1]. Pluripotent stem cells represent a potential therapy for neurological disorders [2]. Improving the effectiveness of hESC-derived neural precursor transplantation therapies has been widely studied [3]. However, teratoma or tumor formation after transplantation remains a major safety concern [4]. Long non-coding RNAs (lncRNAs), defined as longer than 200 nucleotides in length, are suggested to be involved in neural developmental events [5, 6] and in neurological degenerative diseases [7, 8]. Thus, lncRNAs may play important roles in deriving neural lineage cells from hESCs.

Recently, abundant expression of an lncRNA, maternally expressed gene 3 (*Meg3*), which is also called gene trap locus 2 (*Gtl2*), was detected in the mouse nervous system. *Meg3* is expressed in the forebrain in both developing and adult mice [9] as well as in developing corticospinal neurons [10]. This lncRNA is derived from the delta-like homolog 1 gene and the type III iodothyronine deiodinase gene (*Dlk1-Dio3*) imprinted locus, which expresses many non-coding RNAs (ncRNAs) from the maternal chromosome, including the lncRNAs *Meg3/MEG3*, *Rian/MEG8*, and *Mirg*; one C/D box small nucleolar RNA gene cluster; and microRNA (miRNA) clusters. In contrast, three protein-coding genes, *Dlk1*, *Rtl1*, and *Dio3*, are expressed from the paternal chromosome. The correct expression dosages of these ncRNAs and genes are regulated by the two differentially methylated regions of the locus, including the intergenic differentially methylated region (IG-DMR) and the *Meg3/MEG3* DMR [11–13]. Differential methylation of this imprinted locus is required for maintaining the full developmental potential of mouse-induced pluripotent stem cells (miPSCs) [14–16].

Humans possessing imprinting defects in the *DLK1-DIO3* locus suffer from skeletal malformations, developmental delay/mental retardation, tumor development, and even postnatal death [17–24]. Moreover, ncRNAs derived from the *DLK1-DIO3* locus are associated with neurodevelopmental and neurodegenerative disorders. For example, more than 85% of *DLK1-DIO3* locus-derived miRNAs are downregulated in patients with schizophrenia [25], and decreased lncRNA *MEG3* expression occurs in patients with Huntington’s disease [7]. Recently, *DLK1-DIO3* locus-derived lncRNAs were suggested to associate with polycomb repressive complex 2 (PRC2) and to affect genome-wide PRC2 targets *in trans* in mouse embryonic stem cells (mESCs) and human induced pluripotent stem cells (hiPSCs) [26]. PRC2 introduces the specific repressive histone marker H3K27me3 to target regions and suppresses gene expression [27, 28]. Most PRC2 target genes are known for their important roles in developmental processes [28].

To study the correlations between the *DLK1-DIO3*-derived ncRNAs and the differentiation capacity of hESCs toward neural lineages, we classified hESCs into *MEG3*-ON and *MEG3*-OFF on the basis of the expression of *MEG3* and its downstream miRNAs. We found that using hESCs with repressed *DLK1-DIO3*-derived ncRNA expression for neural lineage differentiation produced reduced neural lineage marker expression at different stages and reduced neurite formation.

## Materials and methods

### Ethics statement

This study included the use of human pluripotent stem cells. The derivation of these cell lines and their use in this particular study were approved by the ethics committee of National Taiwan University Hospital and the Internal Research Board of Academia Sinica. Written informed consent was obtained from all subjects involved in this study.

### Human embryonic stem cell culture

The NTU1, NTU3, and H9 hESC lines [29–31] and hiPSC lines [32, 33] were used in this study. These pluripotent stem cells were maintained on murine embryonic fibroblast (MEF) feeders by using serum-free medium (ReproCELL primate and human ESC culture medium; ReproCELL, Kanagawa, Japan). The hESCs were passaged once per week by using a 30-gauge insulin needle or using dispase and collagenase type IV (Gibco, part of Invitrogen, Carlsbad, CA, USA). At 48 hours after passaging, hESC colonies were observed. The medium was refreshed every day. For embryoid body (EB) formation, the hESCs were cultured in suspension in Petri dishes for 5 or 12 days, in accordance with a previously published protocol [29]. On days 5 and 12, EBs were collected for quantitative reverse transcription-polymerase chain reaction (qRT-PCR). The diameters of EBs were quantified by using QCapture Pro 6.0 software (QImaging, Surrey, BC, Canada).

### Quantitative reverse transcription-polymerase chain reaction for long non-coding RNA and mRNA

qRT-PCR was used to quantify relative mRNA levels in the undifferentiated hESCs, EBs, and neural lineage-like cells. Briefly, at a predetermined time point, cells were collected and treated with TRIzol® (Invitrogen) to extract RNA, and samples of cDNA were obtained by using random hexamer primers following the Superscript III kit protocol (Invitrogen). We detected the expression levels of *MEG3* and *MEG8* in hESCs by using the SYBR Green PCR master mix (Kapa Biosystems, Wilmington, MA, USA). PCR was performed in a thermal cycler (LightCycler® 480 II Instrument; Roche, Basel, Switzerland) by using the following program: 50°C for 2 minutes, 95°C for 10 minutes, and 45 cycles of denaturation at 95°C for 15 seconds and annealing and extension at 60°C for 45 seconds. The quantitation of the endoderm, mesoderm, and ectoderm layer-specific transcripts, *SOX17*, *HAND1*, and *PAX6*, respectively, were measured during EB formation by qRT-PCR. The expression levels of neural lineage differentiation-associated genes, which included *PAX6*, *RTN1*, *DLK1*, *SOX11*, *beta-III TUBULIN*, and *MAP2*, were detected in *MEG3*-ON and *MEG3*-OFF hESCs and their differentiated neural lineage-like cells by using the SYBR Green PCR master mix (Kapa Biosystems). The housekeeping gene *GAPDH* was used as a normalization control. The 2^−ΔΔCp^ method was used to quantify the qRT-PCR results. The primer sequences are listed in Additional file 1: Table S1. qRT-PCR results demonstrating consistently detectable and specific signals were presented in the bar charts and subject to statistical analysis. N.D. (not detectable) indicates that the expression of a particular gene in certain samples, if any, was below the sensitivity threshold of qRT-PCR analysis.

### Quantitative reverse transcription-polymerase chain reaction for microRNA

The UPL probe system (Roche) was used to detect the expression of miRNAs, including miR-127-3p, miR-376c, miR-494, miR-495, miR-496, and miR-154. The miRNA detection protocol was based on a previous protocol [34]. PCR was performed in a thermal cycler (LightCycler® 480 II Instrument; Roche). Briefly, a sequence-specific RT primer was used for cDNA synthesis of a specific miRNA in the RT step. Next, a sequence-specific forward primer, a universal reverse primer, and UPL probe 21 were used for amplifying the cDNA. RNU48 was used as an internal control to normalize the miRNA expression levels. The 2^−ΔΔCp^ method was used to quantify the qRT-PCR results. The primer sequences are listed in Additional file 1: Table S1.

### Microarray analysis

#### Sample preparation

We collected and classified NTU1 hESCs into four *MEG3-*ON sublines (NTU 1–1, 1–2, 1–3 and 1–4) and five *MEG3*-OFF sublines (NTU 1–5, 1–6, 1–7, 1–8, and 1–9) on the basis of the *MEG3* expression levels determined by qRT-PCR. We also validated the methylation patterns of the *DLK1-DIO3* locus by bisulfite sequencing. H9 hESCs and EBs were used as positive and negative controls of pluripotency, respectively, for the PluriTest assay but were not used for identifying genes with differential expression between the *MEG3*-ON and *MEG3*-OFF groups.

#### RNA isolation and hybridization

Total RNA was extracted by using TRIzol® reagent (Invitrogen) and purified by using RNeasy® mini kits (Qiagen, Venlo, Limburg, The Netherlands) to obtain 2 μg of RNA. Concentration and quality were assessed by using an electrophoresis bioanalyzer instrument (Agilent Technologies, Santa Clara, CA, USA). The HumanHT-12 version 4.0 Expression BeadChip (Illumina, San Diego, CA, USA) was used on the basis of its up-to-date content derived from the National Center for Biotechnology Information Reference Sequence (NCBI RefSeq) database (Build 36.2, Release 22). Biotin-labeled cRNA prepared from 1.5 μg of RNA from each sample was fragmented, hybridized to individual HT-12 version 4.0 Expression BeadChips by using the direct hybridization assay protocol, and scanned by using a BeadArray Reader (Illumina).

#### Identification of differentially expressed genes

Raw idat microarray files were read into Partek Genomics Suite version 6.5 (Partek, St. Louis, MO, USA) to generate log transformation and quantile normalization. Quality assessment for each array data was also validated by using Partek or R through data distribution and principal component analysis of samples. In total, 47,319 of the 48,206 probe sets were mapped to 21,777 genes, and 14,235 genes had more than one representative probe set. For each redundancy, the probe set with the greatest average expression across all samples was chosen to represent each gene. To identify the most significantly differentially expressed genes (DEGs) between *MEG3*-ON and *MEG3*-OFF hESCs, we selected only the DEGs identified by the following criterion: (i) genes with more than 1.5 times fold change between *MEG3*-ON and *MEG3*-OFF and with significant differential expression value (*P* <0.05) based on Welch’s *t* test (307 genes). (ii) We used the same package in the Bioconductor software package [35] to perform significance analysis of microarray (SAM) [36] analysis for comparisons between two groups (*MEG3*-ON and *MEG3*-OFF) with 1,000 permutations. In total, 314 significant genes with false discovery rate (FDR) of less than 0.05 and d of 1.68 were selected.

On the basis of these criteria, among the two gene lists from Welch’s *t* test (307 genes) and SAM (314 genes), we obtained 114 intersection DEGs for further biological and functional analysis. A hierarchical clustering heatmap of the 114 genes was generated by using ggplot2 [37] packages in the R platform and Euclidean distance and average linkage.

#### Gene Ontology analysis, gene set enrichment assay, and ‘PluriTest’

We used MetaCore™ software (version 6.18, build 65505) from GeneGo (part of Thomson Reuters, New York, NY, USA) to investigate whether the genes showing differential expression between *MEG3*-ON and *MEG3*-OFF hESCs are involved in particular biological processes or diseases. The MetaCore Gene Ontology (GO) biological process or GO disease analysis used the hypergeometric test to select GO biological process gene sets or disease biomarkers enriched in genes that displayed significant differential expression. The *P* value was calculated by using the right-tailed Fisher’s exact test based on the hypergeometric distribution. The corrected *P* values were obtained by FDR calculation based on the Benjamini-Hochberg method [38] of accounting for multiple testing. A background gene list was collected from all 21,777 mapped microarray genes.

Gene Set Enrichment Analysis (GSEA) [39, 40] was performed to determine whether the gene expression levels annotated by the GO term (GO:0007399) associated with nervous system development were specifically enriched in the *MEG3*-ON (NTU 1–1, 1–2, 1–3, and 1–4) or *MEG3*-OFF (NTU 1–5, 1–6, 1–7, 1–8, and 1–9) hESCs; gene sets were identified in which these genes were enriched, together with an associated *P* value calculated by permutation testing. Partial genes from the gene sets were listed in the results. FDR and familywise-error rate (FWER) statistical calculations were performed as described by Subramanian *et al.*[40]*.* Moreover, the hESC gene sets [41] were adopted to test whether the gene sets associated with ESCs could be enriched in the differential expression profiles of the *MEG3*-ON and *MEG3*-OFF groups.

Furthermore, a separate ‘PluriTest’ [42] was applied to evaluate whether the samples possessed or lost pluripotent features by using machine-learning methods. The pluripotency score is a logistic regression model that enables a probability-based choice between the pluripotent and non-pluripotent phenotypic classes based on microarray data. Overall, 12 microarray chips were analyzed in this test—four *MEG3*-ON hESCs (NTU 1–1, 1–2, 1–3, and 1–4), seven *MEG3*-OFF hESCs (NTU 1–5, 1–6, 1–7, 1–8, and 1–9), and two H9 hESCs as the pluripotent controls—and one EB was the pluripotency negative control for the PluriTest. The data were deposited in the NCBI Gene Expression Omnibus (GEO) and are accessible through GEO Series accession number GSE58809.

### Neural lineage differentiation

hESC maintenance and EB formation were described above in the ‘Human embryonic stem cell culture’ section. For neuroectodermal sphere (NES) formation, 4-day-old EBs were cultured in N2 medium containing Dulbecco’s modified Eagle’s medium/F12, NEAA (1X), L-glutamine (2 mM), N2 supplement (1X), sodium pyruvate (1 mM), and basic fibroblast growth factor (20 ng/mL) (Gibco) for 3 days, followed by Matrigel attachment for 3 or 18 days. Half of the N2 medium was refreshed every 48 hours.

### Immunocytochemistry

Cell differentiation was performed in eight-well chamber slides (Millicell EZ slide; Millipore, Billerica, MA, USA). Cells were fixed with 4% paraformaldehyde for 20 minutes and blocked with 2% bovine serum albumin (BSA)/0.1% Triton X-100 in 1X phosphate-buffered saline (PBS) for 5 to 10 minutes. The cells were incubated with primary antibodies at 4°C for 16 hours in PBS containing 2% BSA. The cells were washed and then incubated with fluorescent secondary antibodies (715-485-150 and 711-485-152; Jackson ImmunoResearch, West Grove, PA, USA) for 1 hour. Immunocytochemistry was also performed by using anti-β-III tubulin (MAB1637; Millipore), anti-MAP2 (GTX111679; GeneTex, Irvine, CA, USA), and anti-Ki67 (ab15580; Abcam, Cambridge, UK) antibodies.

### Bisulfite-sequencing analysis

Bisulfite modification was performed by using an EZ DNA Methylation Kit (Zymo Research, Irvine, CA, USA) with genomic DNA extracted from hESCs of different passage numbers. PCR amplification was initiated at 95°C for 5 minutes, followed by 40 cycles of 95°C for 30 seconds, 54°C for 45 seconds, and 72°C for 30 seconds. The following primer sequences were adopted from previous reports: H19 and KvDMR [43, 44], IG-DMR and *MEG3* DMR [19], and *PEG10* DMR [45]. The amplified products were cloned into the pGEM-T Easy vector (Promega, Madison, WI, USA), and 20 clones from each genomic sample were picked for sequencing. The sequences were analyzed by BiQ Analyzer software [46], and only non-clonal sequences are presented.

### Infection of human embryonic stem cells with small interfering RNA and small hairpin RNA lentiviruses

Two knockdown construct types were used in this study. (i) Two *MEG3*-siRNA plasmids with different target sequences and scramble siRNA-GFP plasmids (Abcam Company). The *MEG3*-siRNA plasmids and their target sequences for *MEG3* are as follows: MEG3-451 siRNA-GFP: tgtgttcacctgctagcaaactggagtgt; MEG3-512-siRNA-GFP: actgactctgtcatcacccttatgatgtc. (ii) *MEG3*-shRNA plasmid with the other target sequence and the scrambled shRNA plasmid (OriGene TR30021). The shRNA plasmid and its target sequences for *MEG3* are as follows: TL 320132C MEG3: gagaggttgtttcactggtatctattgca.

Packaging, envelope, and siRNA or shRNA plasmids were transfected into 293 T cells to produce lentiviral particles. Harvested media containing lentivirus were concentrated and used to infect hESCs. The following two infection methods were used in these experiments: (i) Harvested media with lentivirus-containing siRNA plasmid were used to infect hESC clumps (50 to 250 cells per clump). (ii) Harvested media with lentivirus-containing shRNA plasmid were used for single-cell infection.

The infected hESCs were cultured on feeder cells for 4 days and switched to ESC medium containing puromycin (1 μg/mL) for selection. Antibiotic-selected and GFP-positive hESCs were amplified and harvested for qRT-PCR detection.

### Statistics

In most of the experiments using the NTU1 and NTU3 hESC lines, the error bars represent the standard error of the mean (SEM) generated from three biological samples with three technical repeats each. In the experiments using the H9 hESC and hiPSC lines, the error bars represent the SEM generated from one biological sample with three technical repeats. In the *MEG3*-shRNA knockdown experiments, the error bars represent the SEM generated from one biological sample, with three technical repeats each. Student’s *t* test was used for calculating *P* values. *P <0.05 or **P <0.01 was considered to be significant. In the *MEG3*-siRNA knockdown experiments, the error bars represent the SEM generated from one biological sample with three technical repeats. For multiple-group analysis in the *MEG3*-siRNA knockdown experiments, we used one-way analysis of variance followed by Dunnett’s multiple comparisons test and statistics via GraphPad Prism 6 software (GraphPad Software, Inc., La Jolla, CA, USA). Significance was assigned at *P <0.05 and **P <0.01. The data analysis for the arrays is described in the ‘Microarray analysis’ subsection.

## Results

### *MEG3*-OFF human embryonic stem cells have significantly repressed expression of *DLK1-DIO3*-derived long non-coding RNAs and microRNAs

**Figure 1:**
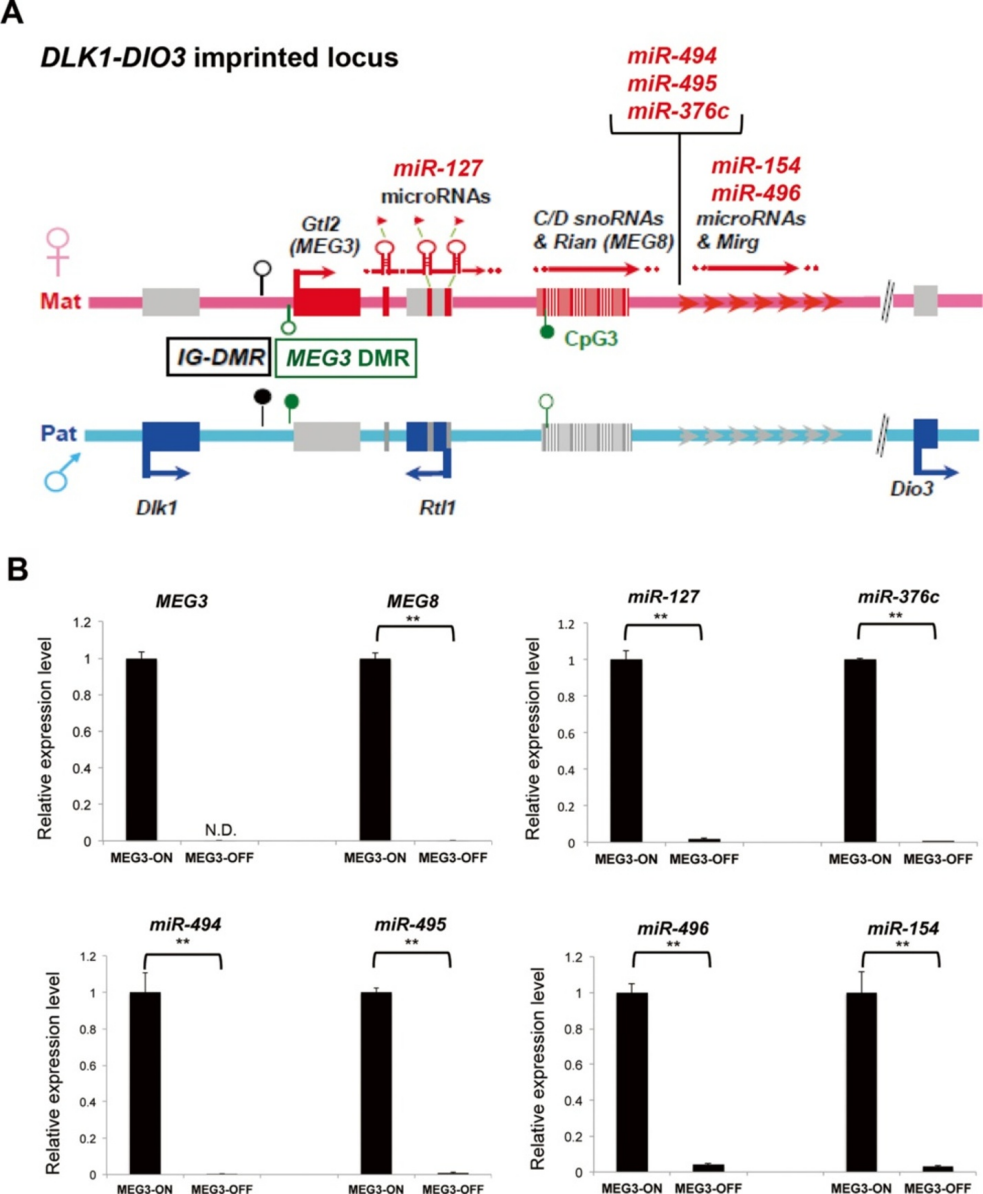
**Classification of** ***MEG3*** **-ON and** ***MEG3*** **-OFF human embryonic stem cells (hESCs) by detecting the expression of the** ***DLK1-DIO3*** **locus-derived non-coding RNAs (ncRNAs). (A)** The *DLK1-DIO3* imprinted locus, which is highly conserved between mice and humans, including clusters of maternally expressed functional ncRNAs, which are marked in red. The human homologs of the *Gtl2* and *Rian* mice genes are *MEG3* and *MEG8*, respectively. Lollipops with closed circles represent methylated CpG regions, and open circles represent unmethylated CpG regions. Mat, maternal chromosome; Pat, paternal chromosome. **(B)** The hESCs with high expression levels of imprinted long non-coding RNAs (lncRNAs) (*MEG3* and *MEG8*) and of several imprinted microRNAs (miRNAs) from the *DLK1-DIO3* locus (miR-127-3p, miR-154, miR-376c, miR-495, miR-494, and miR-496) were classified as *MEG3*-ON hESCs. The hESCs without detectable *MEG3* expression accompanied by significant repression of other ncRNAs from the same locus were classified as *MEG3*-OFF hESCs. *GAPDH* was used as an internal control for mRNA expression analysis, and RNU48 was used as an internal control for miRNA expression analysis. The quantitation of lncRNA and miRNA expression was performed by using the 2^−ΔΔCp^ method. Error bars represent the standard error of the mean generated from three biological repeats. **P <0.01 with respective *MEG3*-ON groups by Student’s *t* test. *DLK1-DIO3*, delta-like homolog 1 gene and the type III iodothyronine deiodinase gene; *Gtl2*, gene trap locus 2; IG-DMR, intergenic differentially methylated region; *MEG3*, maternally expressed gene 3; N.D., not detectable.

The *DLK1-DIO3* imprinted locus is highly conserved between mice and humans (Figure 1A). In this study, we observed that the *DLK1-DIO3* imprinted locus was much more susceptible to hypermethylation than other imprinted loci in both hESC lines and hiPSC lines (Additional file 2: Figure S1). Our miRNA expression profile, which was analyzed by using the Nanostring nCounter™ analysis system (Human miRNA Panel version 2) [47], further confirmed that 37 out of 39 of the most significantly downregulated miRNAs in later-passage hESCs were derived from the *DLK1-DIO3* locus (Additional file 3: Table S2). We collected hESCs and detected their expression levels of *DLK1-DIO3*-derived ncRNAs, including two lncRNAs, *MEG3* (*Gtl2* in mice) and *MEG8* (*Rian* in mice), and several miRNAs from different regions of this locus (Figure 1A). Based on the qRT-PCR results, the hESCs with higher *MEG3* and *MEG8* expression were classified as *MEG3*-ON hESCs, in which several miRNAs from this locus, miR-127-3p, miR-154, miR-376c, miR-494, miR-495, and miR-496, were also abundantly expressed (Figure 1B). In contrast, hESCs sublines in which *MEG3* expression was below the detection threshold of qRT-PCR platform, accompanied by significantly reduced expression of downstream *MEG8* and miRNAs, were classified as *MEG3-*OFF hESCs (Figure 1B). The pluripotency profiles displayed no significant differences in pluripotency-associated gene expression levels between these two hESC sublines on the basis of both GSEA and ‘PluriTest’ (Additional file 4: Figure S2). Thus, *MEG3*-ON and *MEG3*-OFF hESC sublines can serve as a paired study model to illuminate the potential role of *DLK1-DIO3*-derived ncRNAs in hESC-associated research and preclinical model.

### Embryoid bodies derived from *MEG3*-OFF human embryonic stem cells showed unusual morphologies and decreased expression of the ectodermal and neural stem cell marker *PAX6*

**Figure 2:**
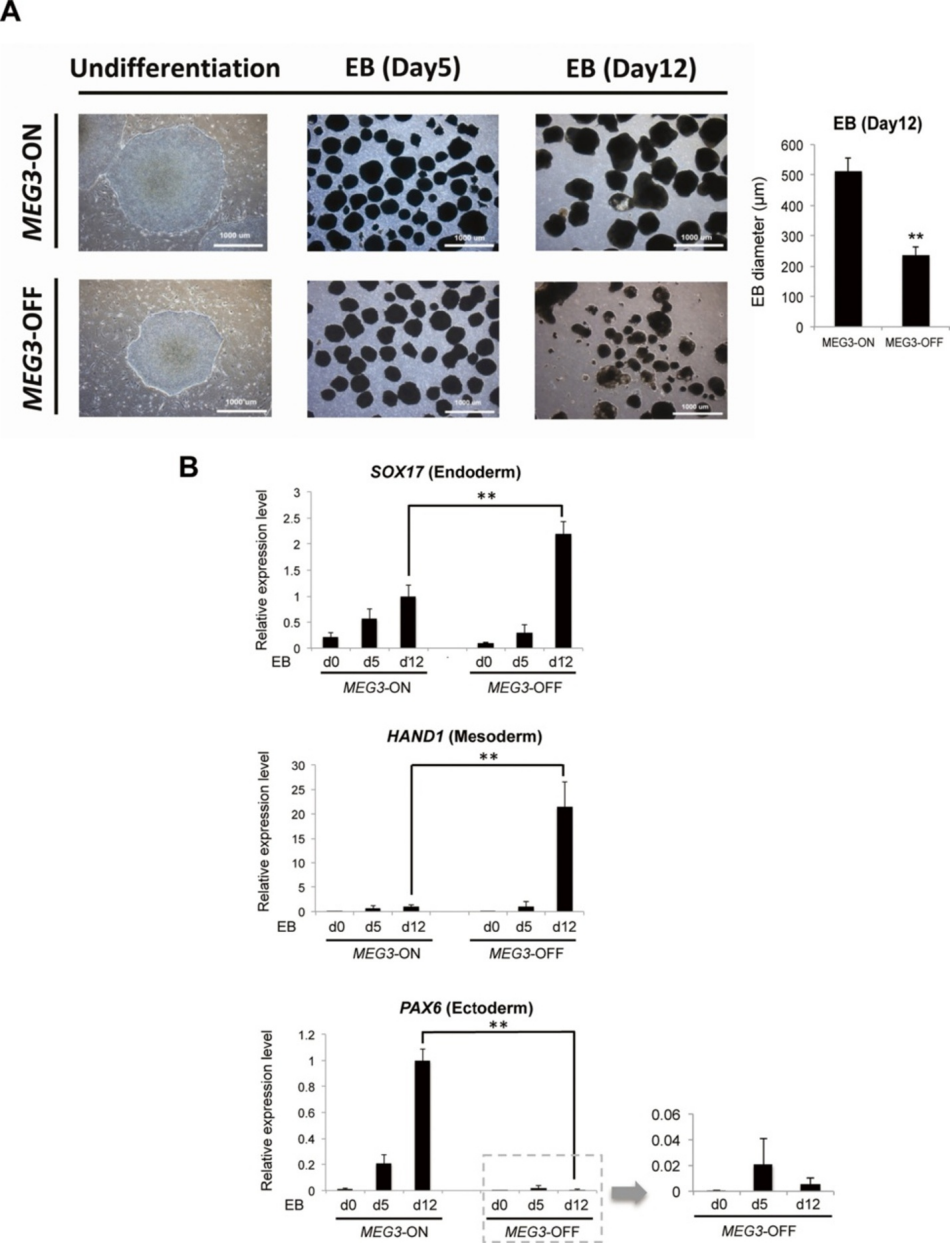
**Twelve**-**day-old embryoid bodies (EBs) differentiated from** ***MEG3*** **-OFF human embryonic stem cells (hESCs) displayed abnormal morphologies and expression levels of developmentally regulated genes. (A)** Day 12 EBs that were differentiated from *MEG3*-OFF NTU1 hESCs were smaller and not well bordered in structure than EBs differentiated from *MEG3*-ON NTU1 hESCs. Scale bars, 1,000 μm. The bar chart illustrated the differences in diameters of the 12-day-old EBs derived from *MEG3*-ON and *MEG3*-OFF hESC sublines. Error bars represent the standard error of the mean (SEM) generated from three biological samples with 20 to 40 EBs in each group. **P <0.01 with respective *MEG3*-ON groups by Student’s *t* test*.* **(B)** Day 12 EBs differentiated from *MEG3*-OFF hESCs displayed unusual expression levels of developmentally regulated genes, including higher expression levels of endoderm- and mesoderm-related genes (*SOX17* and *HAND1*, respectively) and low but detectable expression levels of an ectoderm-related gene (*PAX6*). ‘d’ represents the day of EB formation. *GAPDH* was used as an internal control for mRNA expression analysis. The quantitation of mRNA expression was performed by using the 2^−ΔΔCp^ method. Error bars represent the SEM generated from three biological samples with three technical repeats each. **P <0.01 compared with the corresponding *MEG3*-ON groups by Student’s *t* test. *MEG3*, maternally expressed gene 3.

To investigate the *MEG3*-ON and *MEG3*-OFF hESC differentiation capacities, we began by directly differentiating *MEG3-*ON and *MEG3*-OFF NTU1 hESCs into EBs. No obvious morphological differences were observed between the undifferentiated *MEG3*-ON and *MEG3*-OFF hESCs or between their respective 5-day-old EB derivatives (Figure 2A). However, in prolonged culture up to day 12 after EB formation, the EBs derived from *MEG3*-OFF hESCs were significantly smaller (*P* <0.01), and most of these EBs were not well bordered (Figure 2A).

Moreover, specific markers from the three embryonic germ layers were differentially expressed in 12-day-old EBs (Figure 2B). In *MEG3*-OFF EBs, the endoderm-specific marker *SOX17* and the mesoderm-specific marker *HAND1* were overexpressed, whereas the ectoderm-specific marker *PAX6*, which is also a neural stem cell marker, was only weakly expressed, which may reduce the neural differentiation potential or other differentiation abnormality from *MEG3-*OFF hESC sublines.

### Undifferentiated *MEG3*-OFF human embryonic stem cells **(**hESCs) displayed a different transcriptome profile from *MEG3-*ON hESCs, particularly in the neural lineage-related gene subset

**Figure 3:**
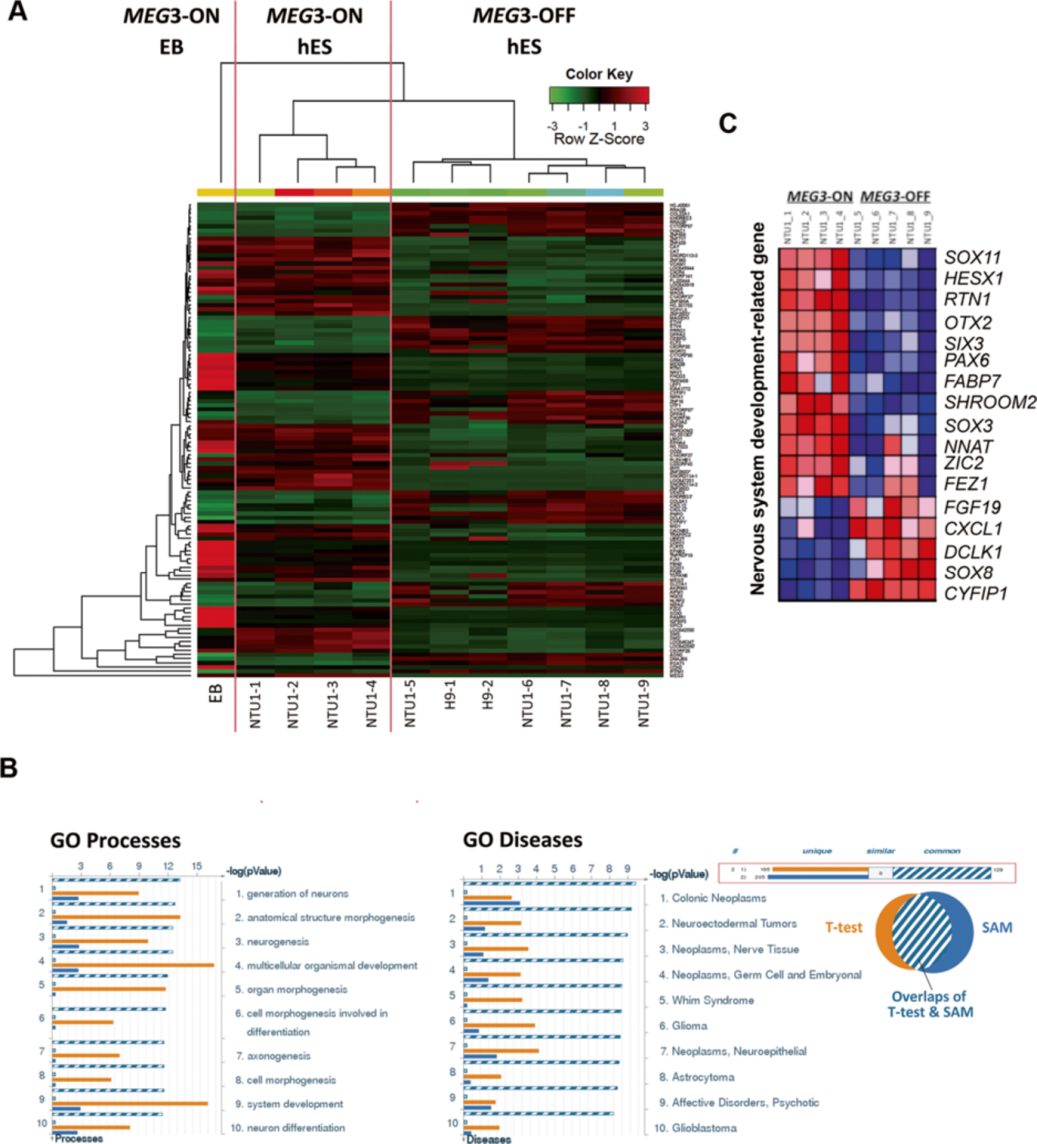
***MEG3*** **-OFF human embryonic stem cells (hESCs) displayed different transcriptome profiles, particularly in genes related to neural lineage. (A)** In total, 114 genes displayed significant differential expression between *MEG3*-ON and *MEG3*-OFF hESCs. The embryoid body (EB) sample differentiated from ‘*MEG3*-ON’ hESCs was used as a reference for differentiated hESCs. The heatmap represents the most significant differentially expressed genes selected by the intersection indicated by Welch’s *t* test (*P* <0.05; fold change >1.5) and significance analysis of microarrays (SAM) (false discovery rate <0.05). The heatmap displays differentially expressed genes that can be used to distinguish between the *MEG3*-ON and *MEG3*-OFF cell states. The green-to-red colors of the heatmap are linearly mapped to the Z-scores, which range from −3 to 3. **(B)** *MEG3*-ON and *MEG3*-OFF hESCs showed differences in the expression levels of many genes that correlate with neural lineage development and with different tumor types. This analysis was performed by using MetaCore software (GeneGo), which includes developmental processes 1,317 Gene Ontology (GO) terms in Biological Process for GO process testing and literature-based biomarkers of clinical diseases for GO disease testing. **(C)** A partial gene list of subset of genes related to ‘nervous system development (GO:0007399)’ was clearly shown to be differentially expressed between undifferentiated *MEG3*-ON and *MEG3*-OFF hESCs by using GSEA. In this heatmap, expression values are represented as colors, with the range of colors (red, pink, light blue, and dark blue) indicating the range of expression values (high, moderate, low, and lowest, respectively). *MEG3*, maternally expressed gene 3.

To further investigate the cellular properties of *MEG3*-ON and *MEG3*-OFF hESCs, we conducted a transcriptome analysis by using cDNA microarrays. We generated a hierarchical clustering heatmap of the 114 genes with significant differential expression between undifferentiated *MEG3*-ON and *MEG3*-OFF hESCs. These 114 DEGs were the intersection of the two gene lists analyzed by Welch’s *t* test (gene selection criteria: *P* value <0.05 and a fold change >1.5) and by SAM (gene selection criterion: FDR <0.05) (Figure 3A). To further examine whether these DEGs were related to neural lineages, MetaCore GO biological process and GO disease analysis were used to analyze the 114 DEGs. Through the GO biological process analysis, many of the DEGs were classified as developmentally regulated genes belonging to neural lineage differentiation, including genes associated with ‘generation of neurons (GO:0048699; FDR = 1.608 × 10^−8^)’, ‘neurogenesis (GO:0022008; FDR = 1.608 × 10^−8^)’, ‘axonogenesis (GO:0007409; FDR = 1.95 × 10^−8^)’, and ‘neuron differentiation (GO:0030182; FDR = 1.217 × 10^−8^)’ (Figure 3B, left panel). Through the GO disease analysis, deregulation of these DEGs was correlated with many tumor types, and most of these DEGs were neural lineage-associated tumors (Figure 3B, right panel). In addition, a partial subset of genes classified as ‘nervous system development (GO:0007399; FDR = 0.014, familywise-error rate <0.001)’ by GSEA also showed significant differential expression patterns between the *MEG3*-ON and *MEG3*-OFF hESCs (Figure 3C). Among these genes, *PAX6* and *RTN1* are known for their functions as a neural stem cell marker and as a neuronal differentiation-related gene [48], respectively (Figure 3C).

**Figure 4:**
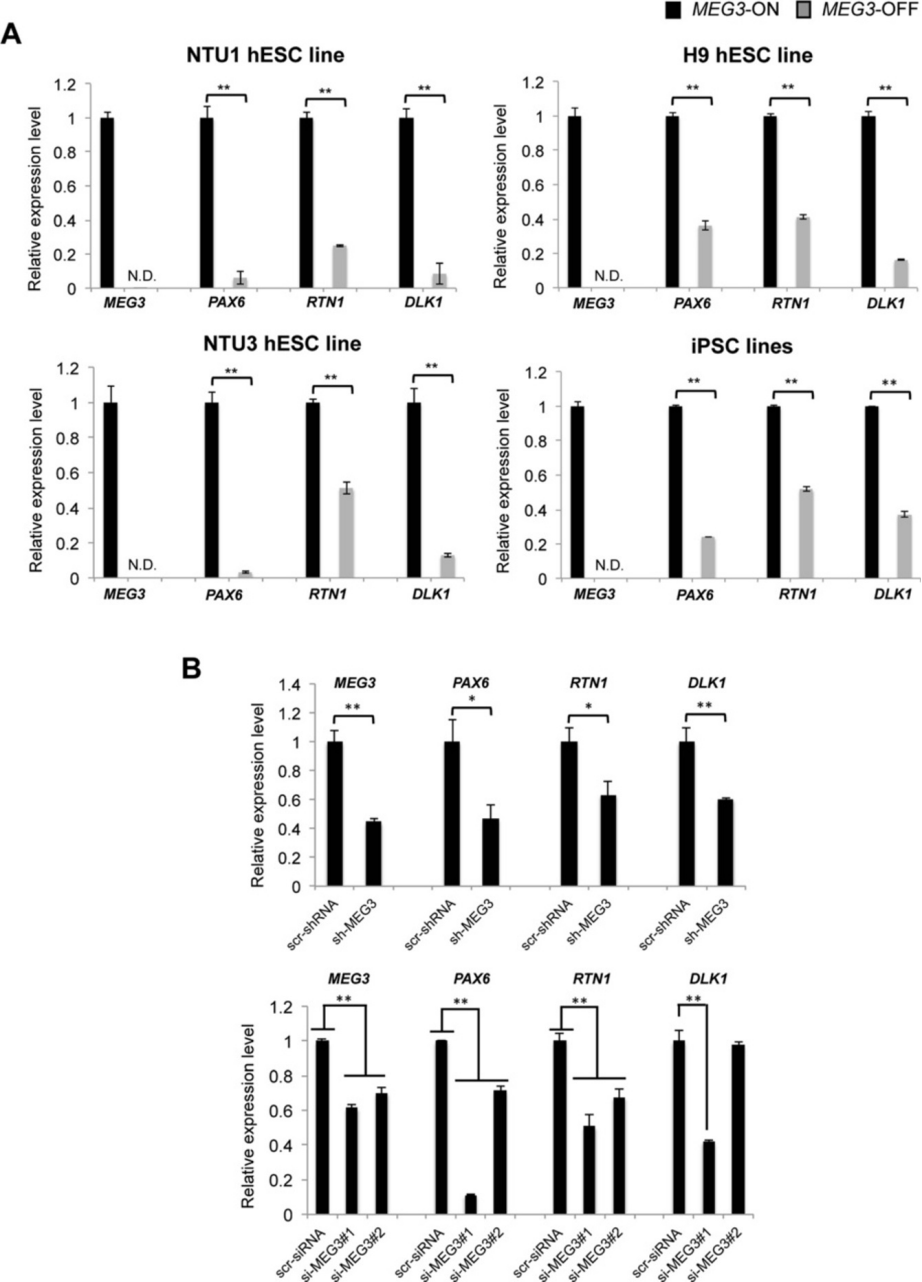
**Associations between the expression of** ***MEG3*** **and neural lineage genes in human embryonic stem cell (hESC) and human induced pluripotent stem cell (hiPSC) lines. (A)** Repression of *MEG3* was consistently correlated with downregulation of *PAX6*, *RTN1*, and *DLK1* in various cell lines. The cell lines where *MEG3* was not detectable, including NTU1, NTU3, H9, and iPSC lines, also displayed lower expression levels of *PAX6*, *RTN1*, and *DLK1*. The mRNA expression was quantified with the 2^−ΔΔCp^ method (using *GAPDH* for normalization). In the NTU1 and NTU3 hESC lines, error bars represent the standard error of the mean (SEM) generated from three biological repeats. In the H9 hESC line and the two iPSC lines, error bars represent the SEM generated from one biological sample with three technical repeats. *P <0.05, **P <0.01 with the corresponding *MEG3*-ON groups by Student’s *t* test*.* N.D., not detectable. **(B)** *MEG3* knockdown assays were conducted via small hairpin RNA (shRNA) and small interfering RNA (siRNA) to examine the association between *MEG3* reduction and the expression levels of neural lineage-related genes in NTU1 hESCs. In the sh-*MEG3* group with *MEG3* reduction, *PAX6*, *RTN1*, and *DLK1* showed downregulated expression (upper panel) compared with the scramble control. *PAX6* and *RTN1* were also downregulated in two si-*MEG3*-treated groups with reduced *MEG3* expression, whereas *DLK1* was reduced in one siRNA-treated group compared with the scramble control (lower panel). The mRNA expression was quantified with the 2^−ΔΔCp^ method (using *GAPDH* for normalization). Error bars represent the SEM generated from one biological sample with three technical repeats each. *P <0.05, **P <0.01 with the corresponding scramble control groups by Student’s *t* test in shRNA experiments; one-way analysis of variance and Dunnett’s multiple comparisons test were used in siRNA experiments, with significance defined as *P <0.05 and **P <0.01. *MEG3*, maternally expressed gene 3.

We validated the association between the levels of *MEG3* expression and *PAX6* and *RTN1* expression by qRT-PCR analysis in paired sublines of three independent hESC lines or in two iPSC lines. The results indicated that *PAX6* and *RTN1* displayed reduced expression in each type of *MEG3*-OFF human pluripotent stem cell line with repressed *MEG3* expression (Figure 4A). Moreover, when *MEG3* expression was reduced in NTU1 hESCs by knockdown via either shRNA or siRNA, *PAX6* and *RTN1* were also downregulated (Figure 4B), suggesting that *MEG3* may directly or indirectly regulate the expression of these genes in hESCs.

Intriguingly, the *DLK1* gene, which is a paternally expressed gene from the same imprinted locus, also showed decreased expression in pluripotent stem cells with repressed *MEG3* expression, in contrast to the expected reciprocal regulation of these two genes (Figure 4A). A *DLK1* function related to neural lineage differentiation was recently observed in mouse and human ESCs [49]. Notably, *DLK1* was also downregulated in two out of three independent *MEG3* knockdown lines: one si-*MEG3* line and one sh-*MEG3* line (Figure 4B). When the two si-*MEG3*-treated hESC lines were compared, the cell line associated with reduced *DLK1* expression was shown to be associated with obvious further *PAX6* downregulation, suggesting that *DLK1* deregulation may be one of the reasons for the reduced expression of these neural lineage-promoting genes (*PAX6* and *RTN1*) in undifferentiated hESCs.

*MEG3* may be directly involved in regulating PRC2 target genes [26], which are often developmentally regulated. Some of the neural progenitor markers, *PAX6*, *CXCR4*, and *SOX21*, which were differentially expressed between *MEG3*-ON and *MEG3*-OFF NTU1 hESCs, were also PRC2 targets subject to EZH2- and SUZ12-mediated H3K27me3 modification [50, 51] (Additional file 5: Figure S3). Therefore, *MEG3* might regulate these neural-progenitor-related genes through a PRC2-mediated mechanism in hESCs.

### *MEG3*-OFF human embryonic stem cell-derived neural lineage-like cells showed lower expression levels of neural lineage markers and reduced neurite formation

**Figure 5:**
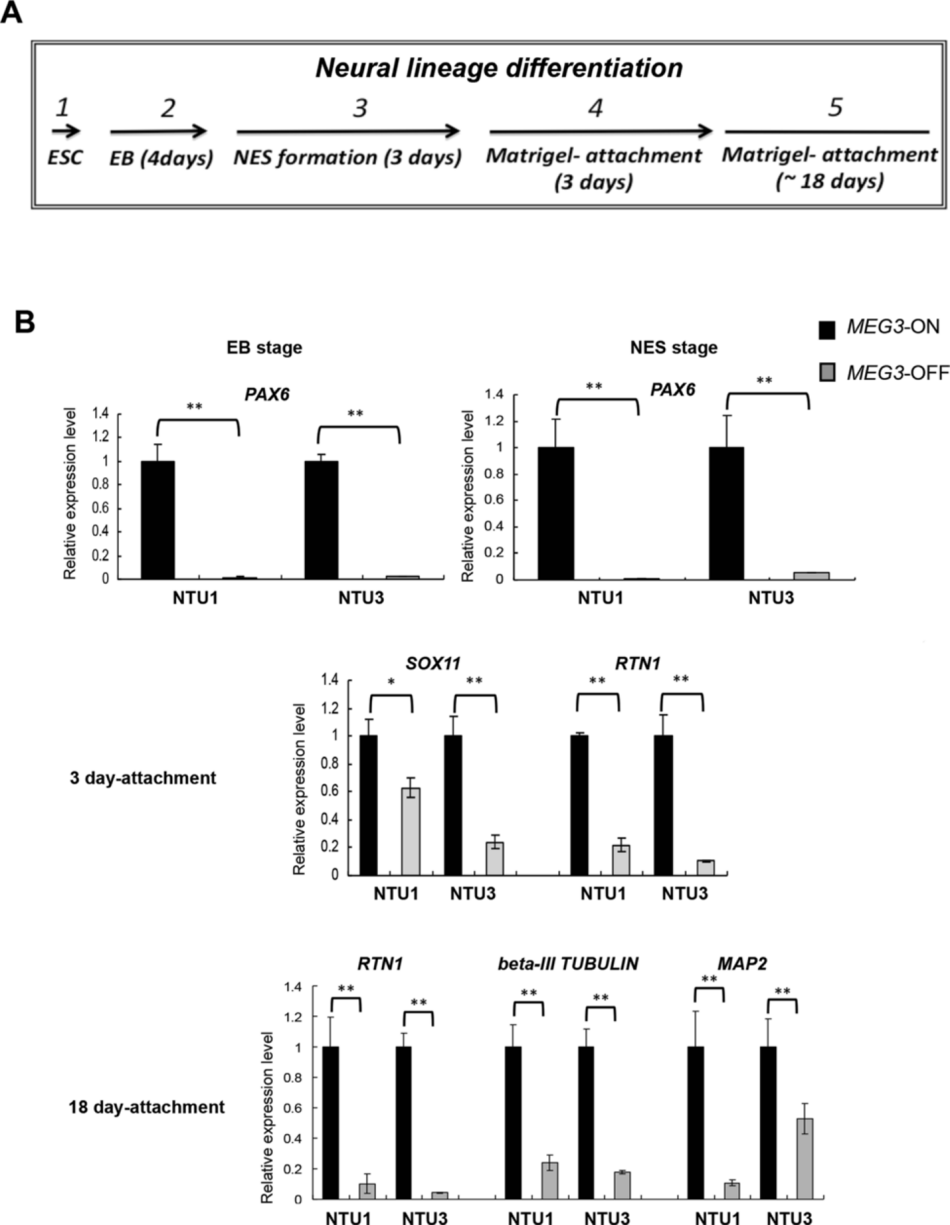
**Neural markers were differentially expressed between** ***MEG3*** **-ON and** ***MEG3*** **-OFF human embryonic stem cell (hESC)-differentiated cells during neural lineage differentiation. (A)** Neural lineage differentiation was conducted from the undifferentiated stage to the 18 day-Matrigel attachment stage in NTU1 and NTU3 hESC lines. **(B)** Expression levels of stage-specific markers were analyzed by quantitative reverse transcription-polymerase chain reaction in *MEG3*-ON and *MEG3*-OFF groups during differentiation. The quantitation of mRNA expression was performed by using the 2^−ΔΔCp^ method (using the housekeeping gene *GAPDH* for normalization). Error bars represent the standard error of the mean generated from three biological samples with three technical repeats each. *P <0.05, **P <0.01 compared with the respective *MEG3*-ON groups by Student’s *t* test. *MEG3*, maternally expressed gene 3; NES, neuroectodermal sphere.

**Figure 6:**
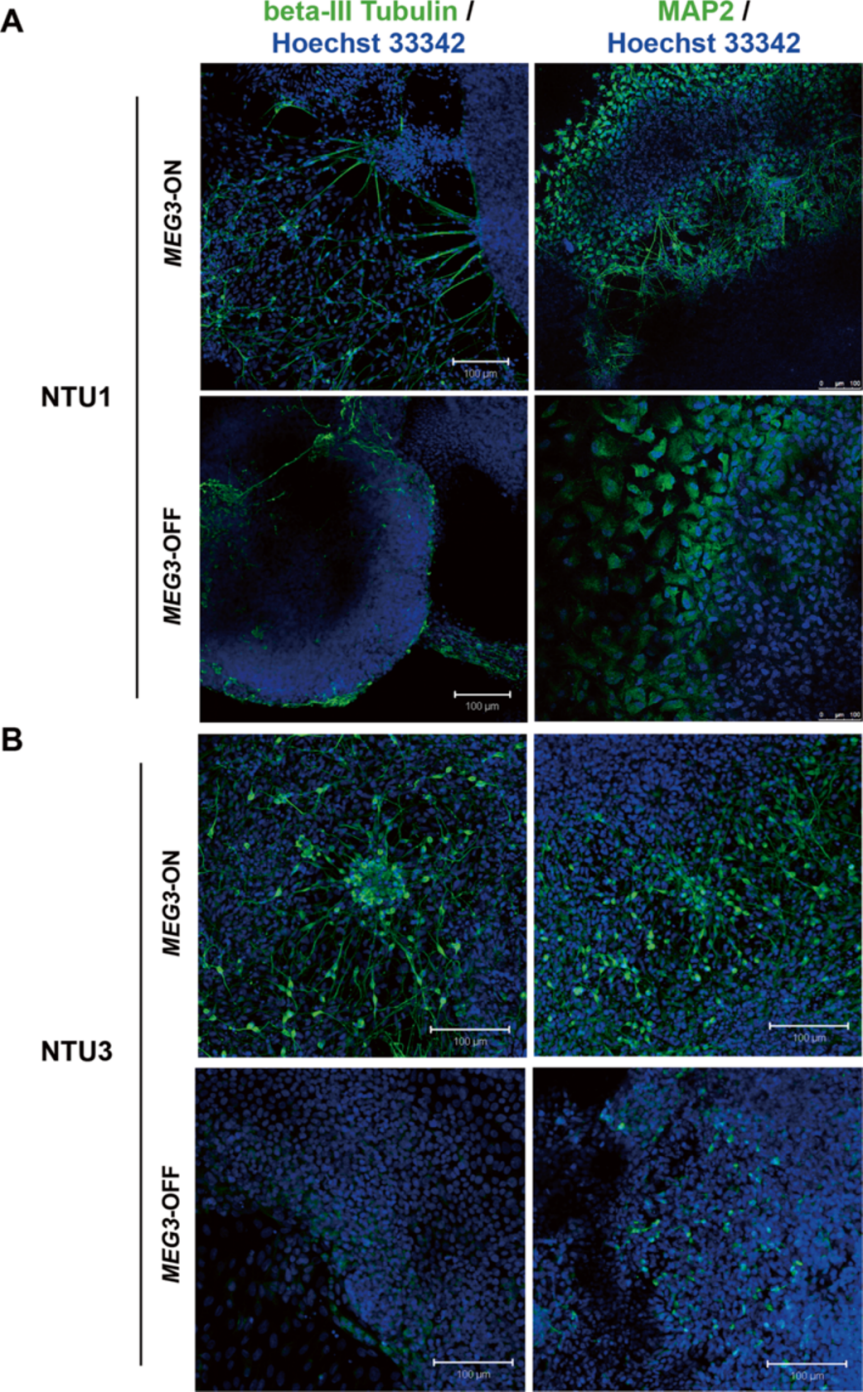
**Neurite formation was reduced in** ***MEG3*** **-OFF human embryonic stem cell (hESC)-differentiated cells compared with cells differentiated from** ***MEG3*** **-ON hESCs.** Immunofluorescent staining was performed with anti-beta-III Tubulin and anti-MAP2 antibodies to examine neurite formation in cells derived from *MEG3*-ON and *MEG3*-OFF hESCs of the NTU1 **(A)** and NTU3 **(B)** cell lines after 18 days of differentiation on Matrigel. Scale bars, 100 μm. *MEG3*, maternally expressed gene 3.

Next, we examined the potential function of ncRNAs derived from the *DLK1-DIO3* imprinted locus in neural lineage differentiation in hESCs. We performed *in vitro* neural lineage differentiation with NTU1 and NTU3 hESC lines (Figure 5A) and investigated the expression levels of several lineage-specific markers, including *PAX6*, *RTN1*, *SOX11*[52], *beta-III Tubulin*, and *MAP2*[53]. At both the EB and the NES stage, the neural stem cell and progenitor cell marker *PAX6* was expressed at lower levels in the *MEG3*-OFF hESC-derived cells (Figure 5B). After 3 days of differentiation on Matrigel, the neuronal precursor and immature neuron marker *SOX11* and *RTN1* that encodes the neuronal differentiation marker RTN-1C were also expressed at dramatically lower levels in the differentiated cells derived from *MEG3*-OFF hESCs (Figure 5B). After 18 days of Matrigel attachment, the neuronal and neurite markers *beta-III Tubulin*, *MAP2*, and *RTN1* were significantly downregulated in the neural lineage-like cells derived from *MEG3*-OFF hESCs (Figure 5B). Most of these neural progenitor and neuronal markers were expressed at low but detectable levels in *MEG3*-OFF hESC-derived cells throughout the differentiation process (Additional file 6: Figure S4). This finding also raised the possibility that the neural lineage differentiation rate of *MEG3*-OFF hESCs might be slower than that of normal hESCs. Furthermore, we analyzed neurite formation capacity by staining with anti-beta-III Tubulin and MAP2 antibodies after 18 days of differentiation on Matrigel (Figure 6). Neural lineage-like cells derived from *MEG3*-ON hESCs displayed obvious neurite formation at this stage. *MEG3*-OFF hESC-derived neural lineage-like cells displayed comparatively reduced neurite formation at the same stage, although some cells were positively stained for beta-III Tubulin and MAP2 (Figure 6A and 6B). The percentage of beta-III Tubulin-positive or MAP2-positive cells differentiated from *MEG3*-OFF hESCs was significantly lower than those from the *MEG3*-ON hESCs (Additional file 7: Figure S5). The numbers of Ki67-positive-stained cells were not obviously different between the *MEG3*-ON and *MEG3*-OFF hESC-derived cells, suggesting those observed differentiation defects seemed not to be linked to the change in cellular proliferation (Additional file 8: Figure S6). Collectively, all of these results suggested that the *MEG3*-ON hESCs with active expression of ncRNAs derived from the *DLK1-DIO3* imprinted locus were associated with a better capacity for neural lineage differentiation.

## Discussion

Selecting high-quality hESC lines has been one of the key issues for stem cell-associated studies and therapies. Imprinting instability has long been a problem for medical applications of pluripotent stem cells, including developmental potential and the risk of cancer formation. The maintenance of differential methylation of the DMRs of imprinted loci is required for the correct dosage of imprinted gene expression, which is suggested to be crucial for ESC function [54, 55]. Accumulating evidence suggests that proper imprinting marks on the mouse *Dlk1-Dio3* locus are important for the biological functions of miPSCs. Aberrant hypermethylation of the *Dlk1-Dio3* locus has been related to the impaired developmental potential of miPSCs. According to the reports by Stadtfeld *et al.*[15, 16] and by Liu *et al.*[14], *Meg3*^*off*^ miPSCs with an aberrantly hypermethylated *Dlk1-Dio3* locus and repressed miRNAs could not achieve full developmental potential for generating full-term mice in a tetraploid complementation assay [14–16]. Ascorbic acid (vitamin C) has been shown to preserve a stable epigenetic state at the mouse *Dlk1-Dio3* locus during somatic cell reprogramming for producing miPSC lines, thereby increasing the success rate of embryos reaching full-term development after tetraploid complementation [16]. These studies indicated that ncRNAs derived from the *Dlk1-Dio3* locus are required for developmental potential in mouse pluripotent stem cells. Moreover, a study by Stadtfeld *et al.* demonstrated that the head tissues of *Meg3*^*off*^ embryos derived from a tetraploid complementation assay with *Meg3*^*off*^ miPSCs displayed dramatic repression of three important neural lineage genes, *Pax6*, *Mash-1*, and *Hes-5*. Their data suggested a possible correlation between the ncRNAs derived from the *DLK1-DIO3* imprinted locus and the expression levels of neural lineage genes. In the present study, we revealed a positive association between the activities of the *DLK1-DIO3* imprinted locus and the differentiation capacity of hESCs for neural lineages on the basis of the following observations: (i) The *MEG3*-OFF hESCs had reduced potential for differentiating into normal EBs, as evidenced by impaired morphologies and by reduced expression of the ectodermal marker *PAX6*. (ii) Many genes involved in nervous system development were differentially expressed between *MEG3*-ON and *MEG3*-OFF undifferentiated hESCs. (iii) Neural lineage-like cells derived from *MEG3-*OFF hESCs showed downregulation of neural lineage markers and reduced neurite formation compared with the *MEG3*-ON groups.

None of the above-mentioned studies can exclude the possibility that unidentified genomic regions other than *DLK1-DIO3* imprinted locus also acquire aberrant epigenetic marks during iPSC reprogramming or *in vitro* ESC culture, which accumulatively or independently cause the functional deficiency of these *MEG3*-OFF-associated pluripotent stem cells. Our *MEG3* knockdown experiments induced downregulation of neural lineage markers, suggesting a certain level of potential causative effect between *MEG3* expression level and the developmental potential of hESCs.

*MEG3* and *DLK1* have been observed to be reciprocally regulated in mouse embryos [11]. However, how these two genes are regulated in mouse and human pluripotent stem cells is not fully understood. Even in mESC lines in which *Meg3* is highly expressed, the expression patterns of *Dlk1* are extremely variable [15]. Based on array data, both *Meg3* and *Dlk1* were shown to be abundantly expressed in one mESC line; however, in three other mESC lines with *Meg3* expression, the *Dlk1* expression level was shown to be lower [15]. Moreover, *Dlk1* expression has also been shown to be low in *Meg3*^*off*^ miPSC lines [15]. In regard to the expression of *DLK1* in hESC lines, the array data from another study demonstrated that it was downregulated in most of the tested *MEG3-*OFF hESC lines [56]. These data imply that the cell type-specific and stage-specific developmental regulation may be the primary factor that controls the net expression level of *DLK1* in undifferentiated hESCs. Kaneko *et al.* did not observe reciprocal correlation of *MEG3* and *DLK1* expression levels between undifferentiated *MEG3*^+^ and *MEG3*^−^ hiPSCs identified in their study. They also did not find the differential PRC2 binding on the *DLK1* promoter when overexpressing *MEG3* in hiPSCs [26]. A *DLK1* function related to neural lineage differentiation was recently observed in mESCs and hESCs. *DLK1*-overexpressing mESCs and hESCs have been shown to promote increased neurogenesis in mESC-derived neural progenitors and hESC-derived neural progenitors, respectively [49]. Interestingly, in our study, the *MEG3*-ON hESCs with increased capacity for neural lineage differentiation displayed relatively higher *DLK1* expression levels compared with the *MEG3*-OFF hESCs. Our study is the first to link these associations between *MEG3* and *DLK1* expression with the capacity for neural lineage differentiation in hESCs.

Although *MEG3* and *DLK1* are reciprocally regulated in E16.6 full mouse embryos by parental origin-specific methylation of their imprinting control region [11], in many mouse pluripotent stem cell lines and hESC lines, including the cells lines in our study, lower *MEG3* expression is surprisingly associated with lower expression of *DLK1*. Because imprinting instability is a frequent phenomenon in *in vitro* culture systems of mouse and human pluripotent stem cells, this finding raises the possibility that amplification of epigenetic abnormalities might be easily observed in *in vitro* culture conditions. We speculate that when maternally expressed ncRNAs derived from the *DLK1-DIO3* locus are silenced, the deregulation of miRNAs from the same locus might affect other downstream factors, including certain epigenetic modifiers. Consistent with this hypothesis, we previously detected a dramatic increase in H3K9me3 and a decrease in H3K4me3 in total protein levels in NTU1 *MEG3*-OFF hESCs (data not shown). These results suggest that the global chromatin state in this cell type may be more condensed, which may further affect the deregulation of genes related to *MEG3* expression levels found in our study, including the *DLK1* gene.

The lncRNA *MEG3* derived from the *DLK1-DIO3* locus possesses tumor suppressor properties in several cancer types, many of which are associated with the nervous system, including pituitary tumors [22], neuroblastomas [57], meningiomas [21], and gliomas [58]. *MEG3* expression is significantly decreased in tumor tissues compared with adjacent normal tissues. *MEG3* overexpression has been shown to promote cell apoptosis in glioma cell lines and inhibit cell proliferation in glioma and meningioma cell lines [21, 58]. We observed that many genes related to various cancer types, primarily those associated with the nervous system, were deregulated in the *MEG3*-OFF hESCs in which *MEG3* and the other downstream ncRNAs were repressed. Therefore, avoiding the use of *MEG3*-OFF hESCs may reduce the potential risk of tumorigenesis from stem cell-based therapies.

Notably, the *RTN1* gene, which is correlated with several neural lineage-type tumors, was dramatically downregulated in *MEG3*-OFF hESCs and in their neural lineage-like derivatives. The *RTN1* gene encodes the RTN1C protein, which is a marker of neuronal differentiation and is known to be downregulated in patients with Alzheimer’s disease and Down syndrome [48]. RTN1C can induce cell apoptosis in neuroectodermal tumors and may be a potential molecular target for therapy [59]. Intriguingly, we observed that *RTN1* was downregulated when *MEG3* was knocked down, suggesting that *MEG3* might participate in the regulation of this gene. Additionally, because *MEG3* and RTN1C can induce apoptosis in cancer cells, determining whether RTN1C is regulated by *MEG3* in cancer models may be worth further investigation.

## Conclusions

For basic and preclinical research, choosing hESC lines with higher expression levels of *DLK1-DIO3* imprinted locus-derived ncRNAs as starting materials, characterized by the expression of the lncRNA *MEG3*, may benefit studies of neural lineages. Additionally, screening for *MEG3*-expressing hESCs may potentially lower the risk of tumorigenesis in stem cell-based therapies.

## Electronic supplementary material

Additional file 1: Table S1: Total primer sequences used in this study. (PDF 84 KB)

Additional file 2: Figure S1: The *DLK1-DIO3* locus was more susceptible to hypermethylation than others in hESC and iPSC lines. **(A)** The IG-DMR and *MEG3* DMR of the *DLK1-DIO3* imprinted locus were two of the first DMRs to be abnormally hypermethylated in NTU1 hESCs subjected to prolonged culture (hypermethylated in the examined hESC samples of P64) in comparison with the *H19* DMR of the *IGF2-H19* imprinted locus and with the *PEG10* DMR and KvDMR from the other two imprinted loci that were differentially methylated in the NTU1 hESCs. P denotes passage numbers. Closed circles represent methylated CpG sites, and open circles represent unmethylated CpG sites. Blue lines between two sets of methylation patterns separate independent bisulfite-sequencing reactions. For identical sequences that cannot be excluded as clonal amplification, we show only one clone and indicate the number of repeats as ‘x N’. **(B)** The *MEG3* DMR of the *DLK1-DIO3* imprinted locus was more susceptible to hypermethylation in a cultured H9 hESC line and in two hiPSC lines in comparison to the *H19* DMR, which was previously thought to be highly susceptible to hypermethylation in cultured pluripotent stem cells. iPS-1 was reprogrammed from granulosa cells, and iPS-2 was reprogrammed from foreskin fibroblasts. Closed circles represent methylated CpG sites, and open circles represent unmethylated CpG sites. Dotted lines between two sets of methylation patterns separate independent bisulfite-sequencing reactions. For identical sequences that cannot be excluded as clonal amplification, we show only one clone and indicate the number of repeats as ‘x N’. *DLK1-DIO3*, delta-like homolog 1 gene and the type III iodothyronine deiodinase gene; DMR, differentially methylated region; hESC, human embryonic stem cell; hiPSC, human induced pluripotent stem cell; IG-DMR, intergenic differentially methylated region; iPSC, induced pluripotent stem cell; *MEG3*, maternally expressed gene 3. (PDF 1 MB)

Additional file 3: Table S2: microRNA profiles of the early and later passage hESCs. Among the 800 miRNAs tested, *DLK1-DIO3* locus derived miRNAs are most dramatically silenced in prolonged cultured hESCs. The value of the two samples represents the counting frequency. *DLK1-DIO3*, delta-like homolog 1 gene and the type III iodothyronine deiodinase gene; hESC, human embryonic stem cell; miRNA, microRNA. (PDF 99 KB)

Additional file 4: Figure S2: No significant difference in pluripotency-associated gene expression was observed between *MEG3*-ON and *MEG3*-OFF hESCs. **(A)** No significant differences in pluripotency-related gene expression were found based on Gene Set Enrichment Analysis (GSEA). **(B)** No significant differences in pluripotency-related gene expression were found based on ‘PluriTest’. Samples A-D consisted of *MEG3*-ON undifferentiated hESCs, sample E consisted of *MEG3*-ON hESC-differentiated EBs, and samples F-L consisted of undifferentiated *MEG3*-OFF hESCs. Samples A-J were derived from the NTU1 hESC line; samples K-L were derived from the H9 hESC line. The area between the red lines indicates the range containing approximately 95% of the tested pluripotent samples. The blue lines indicate the scores observed in approximately 95% of the non-pluripotent samples from other studies. hESC, human embryonic stem cell; *MEG3*, maternally expressed gene 3. (PDF 789 KB)

Additional file 5: Figure S3: *MEG3* may affect the expression of certain PRC2 target genes, including neural lineage-related genes. Significantly differentially expressed genes were identified through significance analysis of microarray (SAM) between *MEG3*-ON and *MEG3*-OFF hESCs (described as Sig. genes in red). *PAX6*, *CXCR4*, and *SOX21* genes were identified among the overlapping clusters of the Sig. genes, EZH2 target genes (GSM327665, GSM831028), SUZ12 target genes (GSM831042, GSM935352), H3K27me3-enriched genes (GSM327663), and neural progenitor markers. hESC, human embryonic stem cell; *MEG3*, maternally expressed gene 3; PRC2, polycomb repressive complex 2. (PDF 362 KB)

Additional file 6: Figure S4: Most neural lineage markers were expressed at lower levels in *MEG3*-OFF hESCs- derived cells throughout differentiation. **(A)** Significantly higher expression levels of *PAX6*, *RTN1*, and *MAP2* were detected in NTU1 and NTU3 *MEG3*-ON hESC-derived cells compared with the *MEG3*-OFF groups throughout the process of neural lineage differentiation. *beta-III TUBULIN* was also significantly upregulated in NTU1 cells derived from *MEG3*-ON hESCs compared with the *MEG3*-OFF groups at the EB stage, at both 3 days and 18 days after attachment; however, *beta-III TUBULIN* was upregulated in the *MEG3*-OFF group at the NES stage. *beta-III TUBULIN* was also detected in NTU3 lines and was upregulated in *MEG3*-ON groups at 18 days after attachment but not at the NES stage or at 3 days after attachment. The quantitation of mRNA expression was performed using the 2^−ΔΔCp^ method (using the housekeeping gene *GAPDH* for normalization). Error bars represent the standard error of the mean generated from two biological samples with three technical repeats each. *P <0.05, **P <0.01 compared with the corresponding *MEG3*-ON groups by Student’s *t* test. **(B)** The melting curves of *PAX6* gene that was significantly expressed at lower but detectable levels in *MEG3*-OFF hESC-differentiated cells at the EB and NES stages. EB, embryoid body; hESC, human embryonic stem cell; *MEG3*, maternally expressed gene 3; NES, neuroectodermal sphere. (PDF 484 KB)

Additional file 7: Figure S5: Quantitation of beta-III Tubulin- or MAP2-positive cells after differentiation from the *MEG3*-ON and *MEG3*-OFF hESCs. The percentage of beta-III Tubulin-positive or MAP2-positive cells differentiated from the NTU1 and NTU3 *MEG3*-OFF hESCs was significantly lower than those from the *MEG3*-ON hESCs. Error bars represent the standard error of the mean generated from two biological samples with three technical repeats each. *P <0.05, **P <0.01 compared with the corresponding *MEG3*-ON groups by Student’s *t* test. hESC, human embryonic stem cell; *MEG3*, maternally expressed gene 3. (PDF 198 KB)

Additional file 8: Figure S6: Ki67 staining for the *MEG3*-ON and *MEG3*-OFF hESC-differentiated neural lineage-like cells after 18 days on Matirgel. The numbers of Ki67-positive-stained cells were not obviously different between the *MEG3*-ON and *MEG3*-OFF hESC-differentiated cells, suggesting that those observed differentiation defects seemed not to be linked to the change in cellular proliferation. Scale bars, 100 μm. hESC, human embryonic stem cell; *MEG3*, maternally expressed gene 3. (PDF 2 MB)

## Abbreviations

BSA: bovine serum albumin
DEG: differentially expressed gene
*DLK1-DIO3*: delta-like homolog 1 gene and the type III iodothyronine deiodinase gene
EB: embryoid body
FDR: false discovery rate
GEO: Gene Expression Omnibus
GO: Gene Ontology
GSEA: Gene Set Enrichment Analysis
*Gtl2*: gene trap locus 2
hESC: human embryonic stem cell
hiPSC: human induced pluripotent stem cell
IG-DMR: intergenic differentially methylated region
lncRNA: long non-coding RNA
*MEG3*: maternally expressed gene 3
mESC: mouse embryonic stem cell
miPSC: mouse-induced pluripotent stem cell
miRNA: microRNA
ncRNA: non-coding RNA
NES: neuroectodermal sphere
PBS: phosphate-buffered saline
PRC2: polycomb repressive complex 2
qRT-PCR: quantitative reverse transcription-polymerase chain reaction
SAM: significance analysis of microarrays
SEM: standard error of the mean
shRNA: small hairpin RNA
siRNA: small interfering RNA.
