# Supplementary material for: Loss of non-coding RNA expression from the DLK1-DIO3 imprinted locus correlates with reduced neural differentiation potential in human embryonic stem cell lines
Source: Stem Cell Res Ther. 2015 Jan 5;6(1):1. doi: 10.1186/scrt535 (PMC4417332; doi:10.1186/scrt535)
Supplement: Supplementary file 1 — Additional file 1: Table S1: Total primer sequences used in this study. (PDF 84 KB) [file 13287_2014_417_MOESM1_ESM.pdf]

Table S1. Total primer sequences used in this study.

Pimer name	Sequence (5' to 3')
	For lncRNA or mRNA detection
MEG3-F	AAGGACCACCTCCTCTCCAT
MEG3-R	AGGAAACCGTGCTCCTAGTG
MEG8-F	TGTCGGAGGATCGTGTCAT
MEG8-R	AATCTTCTAGAGCCCCAGATCC
DLK1-F	ACGATGGCCTCTATGAATGC
DLK1-R	GCAGAAATTGCCTGAGAAGC
PAX6-F	TCACCATGGCAAATAACCTG
PAX6-R	CAGCATGCAGGAGTATGAGG
SOX17-F	ACGCCGAGTTGAGCAAGA
SOX17-R	TCTGCCTCCTCCACGAAG
HAND1-F	TGCCTGAGAAAGAGAACCAG
HAND1-R	ATGGCAGGATGAACAAACAC
SOX11-F	GAGCTGAGCGAGATGATCG
SOX11-R	GAACACCAGGTCGGAGAAGT
RTN1-F	GCAGAAAACCGACGAAGG
RTN1-R	AGGCAGTCCGTGTACTTCTGA
beta-III Tubulin-F	GCAACTACGTGGGCGACT
beta-III Tubulin-R	ATGGCTCGAGGCACGTACT
MAP2-F	CGAACTTTATATTTTACCACTTCCTTG
MAP2-R	CCGTTCATCTGCCATTCTTC
GAPDH-F	AGGGC TGCTT TTAAC TCTGG T
GAPDH-R	CCCCA CTTGA TTTTG GAGGG A
	For miRNA detection
miR-127-3p-F	GGTCGGATCCGTCTGAGC

miR-127-3p-RT	GTTGGCTCTGGTGCAGGGTCCGAGGTATTCGCACC AGAGCCAACAGCCAA
miR-154-F	GCGCTAGGTTATCCGTGTTG
miR-154-RT	GTTGGCTCTGGTGCAGGGTCCGAGGTATTCGCACC AGAGCCAACCGAAGG
miR-376c-F	GGGCGCAACATAGAGGAAATT
miR-376c-RT	GTTGGCTCTGGTGCAGGGTCCGAGGTATTCGCACC AGAGCCAACACGTGG
miR-494-F	ACGTC ATGAA ACATA CACGG GA
miR494-RT	GTTGG CTCTG GTGCA GGGTC CGAGG TATTC GCACC AGAGC CAACG AGGTT
miR-495-F	GCGGAAACAAACATGGTGCA
miR-495-RT	GTTGGCTCTGGTGCAGGGTCCGAGGTATTCGCACC AGAGCCAACAAGAAG
miR-496-F	ACGTC ATGAG TATTA CATGG CC
miR-496-RT	GTTGG CTCTG GTGCA GGGTC CGAGG TATTC GCACC AGAGC CAACG AGATT
RNU48-F	AGTGATGATGACCCCAGGTAA
RNU48-RT	GTTGGCTCTGGTGCAGGGTCCGAGGTATTCGCACC AGAGCCAACGGTCAG
Universal reverse primer	GTGCAGGGTCCGAGGT
	For bisulfilte sequencing
KvDMR-bi-F	TGATGTGTTTATTATTTYGGG
KvDMR-bi-R	CCCTAAAATCCCAAATCCTC
H19 DMR-bi-F	TGTATAGTATATGGGTATTTTTGGAGGTTT
H19 DMR-bi-R	TCCTATAAATATCCTATTCCCAAATAACC
IGDMR(CG4)-bi-F	TTTTATTATTGAATTGGGTTTGTTAGT
IGDMR(CG4)-bi-R	ACAATTCCTACTACAAAATTTCAACA
MEG3 DMR(CG7)-bi-F	TTGTGTTTGAATTTATTTTGTTT
MEG3 DMR(CG7)-bi-R	CCCCAAATTCTATAACAAATTACT

PEG10 DMR-bi-F	GTGTTATGTTTTATAAATAGATAAG
PEG10 DMR-bi- R	AACTCATATACCTCTACAATTC
