# Supplementary material for: Loss of non-coding RNA expression from the DLK1-DIO3 imprinted locus correlates with reduced neural differentiation potential in human embryonic stem cell lines
Source: Stem Cell Res Ther. 2015 Jan 5;6(1):1. doi: 10.1186/scrt535 (PMC4417332; doi:10.1186/scrt535)
Supplement: Supplementary file 2 — Additional file 2: Figure S1: The DLK1-DIO3 locus was more susceptible to hypermethylation than others in hESC and iPSC lines. (A) The IG-DMR and MEG3 DMR of the DLK1-DIO3 imprinted locus were two of the first DMRs to be abnormally hypermethylated in NTU1 hESCs subjected to prolonged culture (hypermethylated in the examined hESC samples of P64) in comparison with the H19 DMR of the IGF2-H19 imprinted locus and with the PEG10 DMR and KvDMR from the other two imprinted loci that were differentially methylated in the NTU1 hESCs. P denotes passage numbers. Closed circles represent methylated CpG sites, and open circles represent unmethylated CpG sites. Blue lines between two sets of methylation patterns separate independent bisulfite-sequencing reactions. For identical sequences that cannot be excluded as clonal amplification, we show only one clone and indicate the number of repeats as ‘x N’. (B) The MEG3 DMR of the DLK1-DIO3 imprinted locus was more susceptible to hypermethylation in a cultured H9 hESC line and in two hiPSC lines in comparison to the H19 DMR, which was previously thought to be highly susceptible to hypermethylation in cultured pluripotent stem cells. iPS-1 was reprogrammed from granulosa cells, and iPS-2 was reprogrammed from foreskin fibroblasts. Closed circles represent methylated CpG sites, and open circles represent unmethylated CpG sites. Dotted lines between two sets of methylation patterns separate independent bisulfite-sequencing reactions. For identical sequences that cannot be excluded as clonal amplification, we show only one clone and indicate the number of repeats as ‘x N’. DLK1-DIO3, delta-like homolog 1 gene and the type III iodothyronine deiodinase gene; DMR, differentially methylated region; hESC, human embryonic stem cell; hiPSC, human induced pluripotent stem cell; IG-DMR, intergenic differentially methylated region; iPSC, induced pluripotent stem cell; MEG3, maternally expressed gene 3. (PDF 1 MB) [file 13287_2014_417_MOESM2_ESM.pdf]

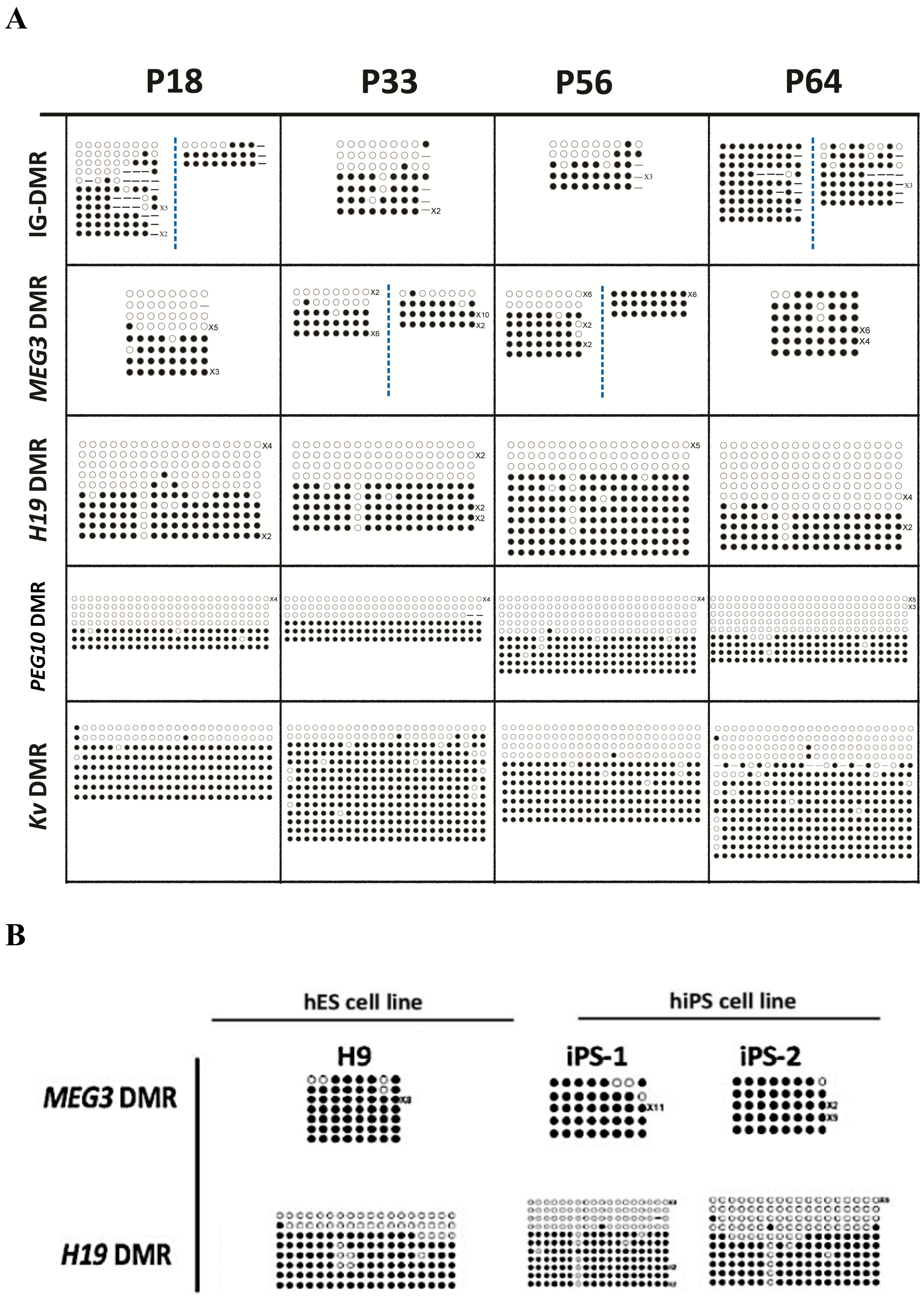

Figure S1. The *DLK1-DIO3* locus was more susceptible to hypermethylation than others in hESC and iPSC lines.

(A) The IG-DMR and *MEG3* DMR of the *DLK1-DIO3* imprinted locus were two of the first DMRs to be abnormally hypermethylated in NTU1 hESCs subjected to prolonged culture (hypermethylated in the examined hESC samples of P64) in comparison with the *H19* DMR of the *IGF2-H19* imprinted locus and with the *PEG10* DMR and KvDMR from the other two imprinted loci that were differentially methylated in the NTU1 hESCs. P denotes passage numbers. Closed circles represent methylated CpG sites, and open circles represent unmethylated CpG sites. Blue lines between two sets of methylation patterns separate independent bisulfite-sequencing reactions. For identical sequences that cannot be excluded as clonal amplification, we show only one clone and indicate the number of repeats as "x N".

(B) The *MEG3* DMR of the *DLK1-DIO3* imprinted locus was more susceptible to hypermethylation in a cultured H9 hESC line and in two hiPSC lines in comparison to the *H19* DMR, which was previously thought to be highly susceptible to hypermethylation in cultured pluripotent stem cells. iPS-1 was reprogrammed from granulosa cells, and iPS-2 was reprogrammed from foreskin fibroblasts. Closed circles represent methylated CpG sites, and open circles represent unmethylated CpG sites. Dotted lines between two sets of methylation patterns separate independent

bisulfite-sequencing reactions. For identical sequences that cannot be excluded as clonal amplification, we show only one clone and indicate the number of repeats as “x N”.
