# Supplementary material for: Loss of non-coding RNA expression from the DLK1-DIO3 imprinted locus correlates with reduced neural differentiation potential in human embryonic stem cell lines
Source: Stem Cell Res Ther. 2015 Jan 5;6(1):1. doi: 10.1186/scrt535 (PMC4417332; doi:10.1186/scrt535)
Supplement: Supplementary file 3 — Additional file 3: Table S2: microRNA profiles of the early and later passage hESCs. Among the 800 miRNAs tested, DLK1-DIO3 locus derived miRNAs are most dramatically silenced in prolonged cultured hESCs. The value of the two samples represents the counting frequency. DLK1-DIO3, delta-like homolog 1 gene and the type III iodothyronine deiodinase gene; hESC, human embryonic stem cell; miRNA, microRNA. (PDF 99 KB) [file 13287_2014_417_MOESM3_ESM.pdf]

Table S2. microRNA profiles of the early and later passage hESCs

Among the 800 miRNAs tested, *DLK1-DIO3* locus derived miRNAs are most dramatically silenced in prolonged cultured hESCs. The value of the two samples represents the counting frequency.

		**sample 1**	**sample 2**		
	**miRNA**	**Early passages**	**Later passages**	**ratio**	***DLK1-DIO3* locus**
1	hsa-miR-409-3p	513.6	0		Yes
2	hsa-miR-381	366.02	0		Yes
3	hsa-miR-323a-3p	326.17	0		Yes
4	hsa-miR-411-5p	214.02	0		Yes
5	hsa-miR-487a	203.69	0		Yes
6	hsa-miR-431-5p	175.65	0		Yes
7	hsa-miR-487b	165.32	0		Yes
8	hsa-miR-370	72.34	0		Yes
9	hsa-miR-409-5p	70.87	0		Yes
10	hsa-miR-379-5p	165.32	4.63	35.70626	Yes
11	hsa-miR-376c	816.13	22.95	35.56122	Yes
12	hsa-miR-136-5p	398.49	12.48	31.93029	Yes
13	hsa-miR-127-3p	520.98	17.72	29.40068	Yes
14	hsa-miR-210	26.59	0		No
15	hsa-miR-543	301.09	12.48	24.1258	Yes
16	hsa-miR-485-3p	299.61	12.48	24.00721	Yes
17	hsa-miR-495	662.65	28.19	23.50656	Yes
18	hsa-miR-376a-3p	1914.1	101.47	18.8637	Yes
19	hsa-miR-376b	289.28	17.72	16.32506	Yes
20	hsa-miR-377-3p	236.15	15.1	15.63907	Yes
21	hsa-miR-154-5p	14.79	0		Yes
22	hsa-miR-337-5p	522.45	41.27	12.65932	Yes
23	hsa-miR-337-3p	311.42	25.57	12.17912	Yes
24	hsa-miR-410	274.52	22.95	11.96166	Yes
25	hsa-miR-758	94.48	9.87	9.572442	Yes
26	hsa-miR-299-5p	134.32	15.1	8.895364	Yes

27	hsa-miR-127-5p	7.41	0		Yes
28	hsa-miR-433	32.5	4.63	7.019438	Yes
29	hsa-miR-654-3p	28.07	4.63	6.062635	Yes
30	hsa-miR-656	87.1	15.1	5.768212	Yes
31	hsa-miR-382-5p	205.16	43.89	4.674413	Yes
32	hsa-miR-432-5p	191.88	41.27	4.649382	Yes
33	hsa-miR-496	8.88	2.02	4.39604	Yes
34	hsa-miR-889	42.83	15.1	2.836424	Yes
35	hsa-miR-299-3p	175.65	70.06	2.507137	Yes
36	hsa-miR-329	32.5	15.1	2.152318	Yes
37	hsa-miR-125b-5p	978.47	593.49	1.648671	No
38	hsa-miR-935	1.51	0		Yes
39	hsa-miR-494	433.91	321.31	1.35044	Yes
40	hsa-miR-188-5p	19.21	15.1	1.272185	No
41	hsa-miR-340-5p	48.73	38.66	1.260476	No
42	hsa-miR-27b-3p	113.66	91	1.249011	No
43	hsa-miR-361-3p	11.84	9.87	1.199595	No
44	hsa-miR-335-5p	113.66	96.23	1.181129	No
45	hsa-miR-24-3p	94.48	80.53	1.173227	No
46	hsa-miR-342-3p	109.24	93.62	1.166845	No
47	ACTB	1707.5	1499.03	1.13907	No
