# Supplementary material for: Loss of non-coding RNA expression from the DLK1-DIO3 imprinted locus correlates with reduced neural differentiation potential in human embryonic stem cell lines
Source: Stem Cell Res Ther. 2015 Jan 5;6(1):1. doi: 10.1186/scrt535 (PMC4417332; doi:10.1186/scrt535)
Supplement: Supplementary file 4 — Additional file 4: Figure S2: No significant difference in pluripotency-associated gene expression was observed between MEG3-ON and MEG3-OFF hESCs. (A) No significant differences in pluripotency-related gene expression were found based on Gene Set Enrichment Analysis (GSEA). (B) No significant differences in pluripotency-related gene expression were found based on ‘PluriTest’. Samples A-D consisted of MEG3-ON undifferentiated hESCs, sample E consisted of MEG3-ON hESC-differentiated EBs, and samples F-L consisted of undifferentiated MEG3-OFF hESCs. Samples A-J were derived from the NTU1 hESC line; samples K-L were derived from the H9 hESC line. The area between the red lines indicates the range containing approximately 95% of the tested pluripotent samples. The blue lines indicate the scores observed in approximately 95% of the non-pluripotent samples from other studies. hESC, human embryonic stem cell; MEG3, maternally expressed gene 3. (PDF 789 KB) [file 13287_2014_417_MOESM4_ESM.pdf]

Figure S2

A

GSEA for the correlation of MEG3 groups and ES sets

Table: GSEA Results Summary

Dataset	all gene.class.cls#MEG3up_versus_MEG3down
Phenotype	class.cls#MEG3up_versus_MEG3down
Upregulated in class	MEG3down
GeneSet	BENPORATH_ES_1
Enrichment Score (ES)	-0.3435752
Normalized Enrichment Score (NES)	-1.030656
Nominal p-value	0.36567163
FDR q-value	0.38619402
FWER p-Value	0.196

Table: GSEA Results Summary

Dataset	all gene.class.cls#MEG3up_versus_MEG3down
Phenotype	class.cls#MEG3up_versus_MEG3down
Upregulated in class	MEG3down
GeneSet	BENPORATH_ES_2
Enrichment Score (ES)	-0.43608865
Normalized Enrichment Score (NES)	-1.1982368
Nominal p-value	0.13369964
FDR q-value	0.14468865
FWER p-Value	0.073

B

Model-Based Multi-Class Pluripotency Score

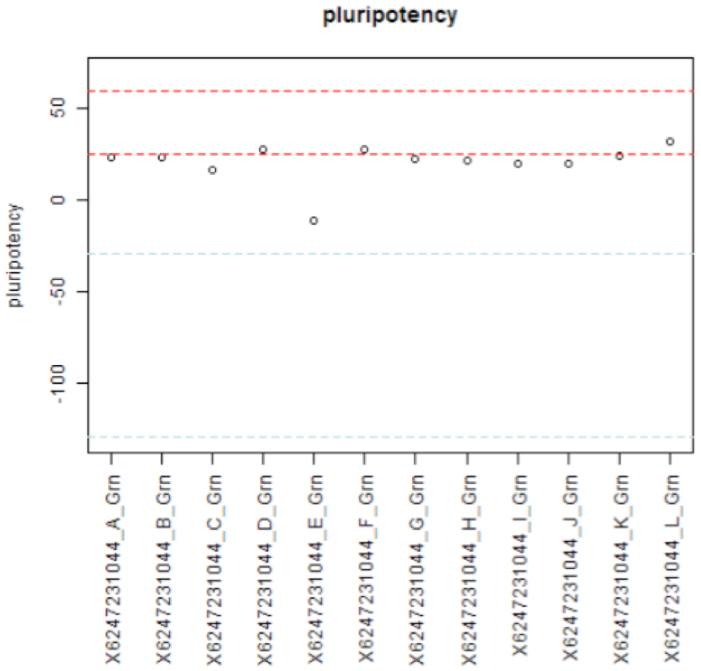

Figure S2. No significant difference in pluripotency-associated gene expression was observed between *MEG3*-ON and *MEG3*-OFF hESCs.

(A) No significant differences in pluripotency-related gene expression were found based on Gene Set Enrichment Analysis (GSEA).

(B) No significant differences in pluripotency-related gene expression were found based on "PluriTest". Samples A-D consisted of *MEG3*-ON undifferentiated hESCs, sample E consisted of *MEG3*-ON hESC-differentiated EBs, and samples F-L consisted of undifferentiated *MEG3*-OFF hESCs. Samples A-J were derived from the NTU1 hESC line; samples K-L were derived from the H9 hESC line. The area between the red lines indicates the range containing approximately 95 percent of the tested pluripotent samples. The blue lines indicate the scores observed in approximately 95 percent of the non-pluripotent samples from other studies.
