# Supplementary material for: Loss of non-coding RNA expression from the DLK1-DIO3 imprinted locus correlates with reduced neural differentiation potential in human embryonic stem cell lines
Source: Stem Cell Res Ther. 2015 Jan 5;6(1):1. doi: 10.1186/scrt535 (PMC4417332; doi:10.1186/scrt535)
Supplement: Supplementary file 5 — Additional file 5: Figure S3: MEG3 may affect the expression of certain PRC2 target genes, including neural lineage-related genes. Significantly differentially expressed genes were identified through significance analysis of microarray (SAM) between MEG3-ON and MEG3-OFF hESCs (described as Sig. genes in red). PAX6, CXCR4, and SOX21 genes were identified among the overlapping clusters of the Sig. genes, EZH2 target genes (GSM327665, GSM831028), SUZ12 target genes (GSM831042, GSM935352), H3K27me3-enriched genes (GSM327663), and neural progenitor markers. hESC, human embryonic stem cell; MEG3, maternally expressed gene 3; PRC2, polycomb repressive complex 2. (PDF 362 KB) [file 13287_2014_417_MOESM5_ESM.pdf]

Figure S3

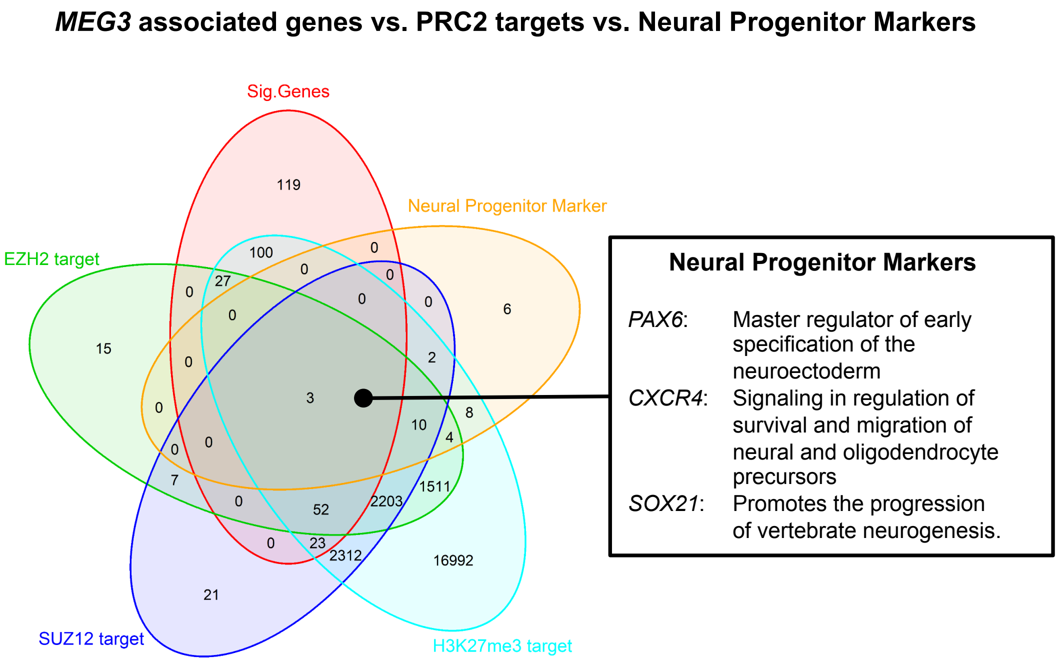

Figure S3. *MEG3* may affect the expression of certain PRC2 target genes, including neural-lineage-related genes.

Significantly differentially expressed genes were identified through significance analysis of microarray (SAM) between *MEG3*-ON and *MEG3*-OFF hESCs (described as Sig. genes in red). *PAX6*, *CXCR4* and *SOX21* genes were identified among the overlapping clusters of the Sig. genes, EZH2 target genes (GSM327665, GSM831028), SUZ12 target genes (GSM831042, GSM935352), H3K27me3 enriched genes (GSM327663) and neural progenitor markers.
