# Supplementary material for: Loss of non-coding RNA expression from the DLK1-DIO3 imprinted locus correlates with reduced neural differentiation potential in human embryonic stem cell lines
Source: Stem Cell Res Ther. 2015 Jan 5;6(1):1. doi: 10.1186/scrt535 (PMC4417332; doi:10.1186/scrt535)
Supplement: Supplementary file 6 — Additional file 6: Figure S4: Most neural lineage markers were expressed at lower levels in MEG3-OFF hESCs- derived cells throughout differentiation. (A) Significantly higher expression levels of PAX6, RTN1, and MAP2 were detected in NTU1 and NTU3 MEG3-ON hESC-derived cells compared with the MEG3-OFF groups throughout the process of neural lineage differentiation. beta-III TUBULIN was also significantly upregulated in NTU1 cells derived from MEG3-ON hESCs compared with the MEG3-OFF groups at the EB stage, at both 3 days and 18 days after attachment; however, beta-III TUBULIN was upregulated in the MEG3-OFF group at the NES stage. beta-III TUBULIN was also detected in NTU3 lines and was upregulated in MEG3-ON groups at 18 days after attachment but not at the NES stage or at 3 days after attachment. The quantitation of mRNA expression was performed using the 2−ΔΔCp method (using the housekeeping gene GAPDH for normalization). Error bars represent the standard error of the mean generated from two biological samples with three technical repeats each. *P <0.05, **P <0.01 compared with the corresponding MEG3-ON groups by Student’s t test. (B) The melting curves of PAX6 gene that was significantly expressed at lower but detectable levels in MEG3-OFF hESC-differentiated cells at the EB and NES stages. EB, embryoid body; hESC, human embryonic stem cell; MEG3, maternally expressed gene 3; NES, neuroectodermal sphere. (PDF 484 KB) [file 13287_2014_417_MOESM6_ESM.pdf]

Figure S4

A

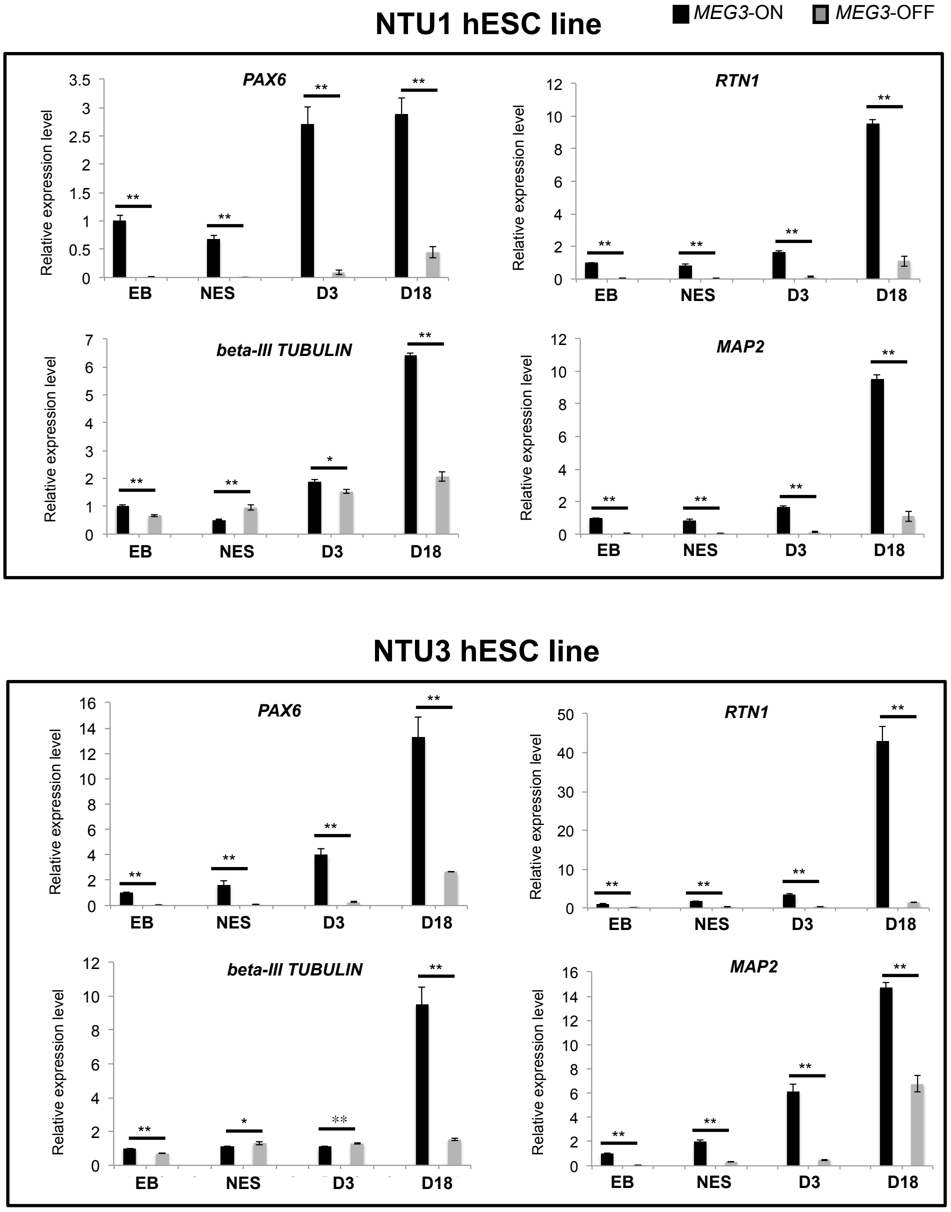
NTU1 hESC line
MEG3-ON
MEG3-OFF
PAX6
RTN1
beta-III TUBULIN
MAP2
Relative expression level
EB
NES
D3
D18
NTU3 hESC line

B

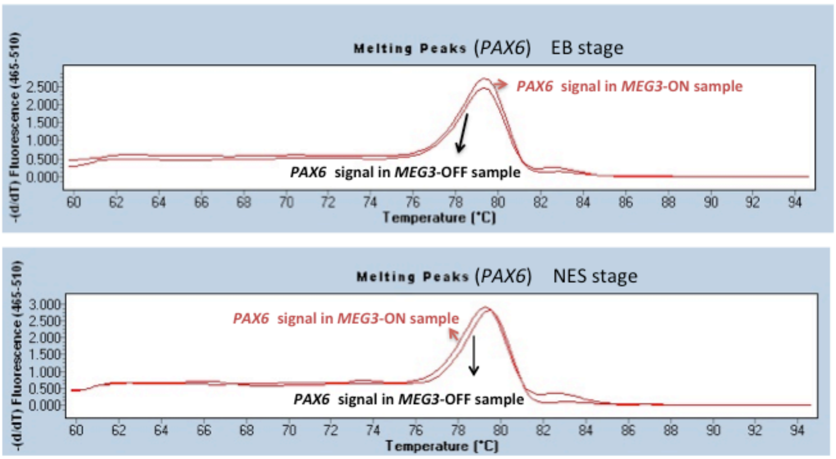
Melting Peaks (PAX6) EB stage
PAX6 signal in MEG3-ON sample
PAX6 signal in MEG3-OFF sample
-(d/dT) Fluorescence (465-510)
Temperature (°C)
Melting Peaks (PAX6) NES stage

Figure S4. Most neural lineage markers were expressed at lower levels in *MEG3*-OFF hESCs- derived cells throughout differentiation.

(A) Significantly higher expression levels of *PAX6*, *RTN1* and *MAP2* were detected In NTU1 and NTU3 *MEG3*-ON hESC-derived cells compared with the *MEG3*-OFF groups throughout the process of neural lineage differentiation. *beta-III TUBULIN* was also significantly upregulated in NTU1 cells derived from *MEG3*-ON hESCs compared with the *MEG3*-OFF groups at the EB stage, at both 3 days and 18 days after attachment; however, *beta-III TUBULIN* was upregulated in the *MEG3*-OFF group at the NES stage. *beta-III TUBULIN* was also detected in NTU3 lines and was upregulated in *MEG3*-ON groups at 18 days after attachment but not at the NES stage or at 3 days after attachment. The quantitation of mRNA expression was performed using the $2^{-\Delta\Delta Cp}$ method (using the housekeeping gene *GAPDH* for normalization). Error bars represent the SEM generated from 2 biological samples with 3 technical repeats each. *P<0.05, ** P<0.01 compared with the corresponding *MEG3*-ON groups by Student's *t*-test.

(B) The melting curves of *PAX6* gene that was significantly expressed at lower but detectable levels in *MEG3*-OFF hESC-differentiated cells at the EB and NES stages.
