# Supplementary material for: Loss of non-coding RNA expression from the DLK1-DIO3 imprinted locus correlates with reduced neural differentiation potential in human embryonic stem cell lines
Source: Stem Cell Res Ther. 2015 Jan 5;6(1):1. doi: 10.1186/scrt535 (PMC4417332; doi:10.1186/scrt535)
Supplement: Supplementary file 7 — Additional file 7: Figure S5: Quantitation of beta-III Tubulin- or MAP2-positive cells after differentiation from the MEG3-ON and MEG3-OFF hESCs. The percentage of beta-III Tubulin-positive or MAP2-positive cells differentiated from the NTU1 and NTU3 MEG3-OFF hESCs was significantly lower than those from the MEG3-ON hESCs. Error bars represent the standard error of the mean generated from two biological samples with three technical repeats each. *P <0.05, **P <0.01 compared with the corresponding MEG3-ON groups by Student’s t test. hESC, human embryonic stem cell; MEG3, maternally expressed gene 3. (PDF 198 KB) [file 13287_2014_417_MOESM7_ESM.pdf]

Figure S5

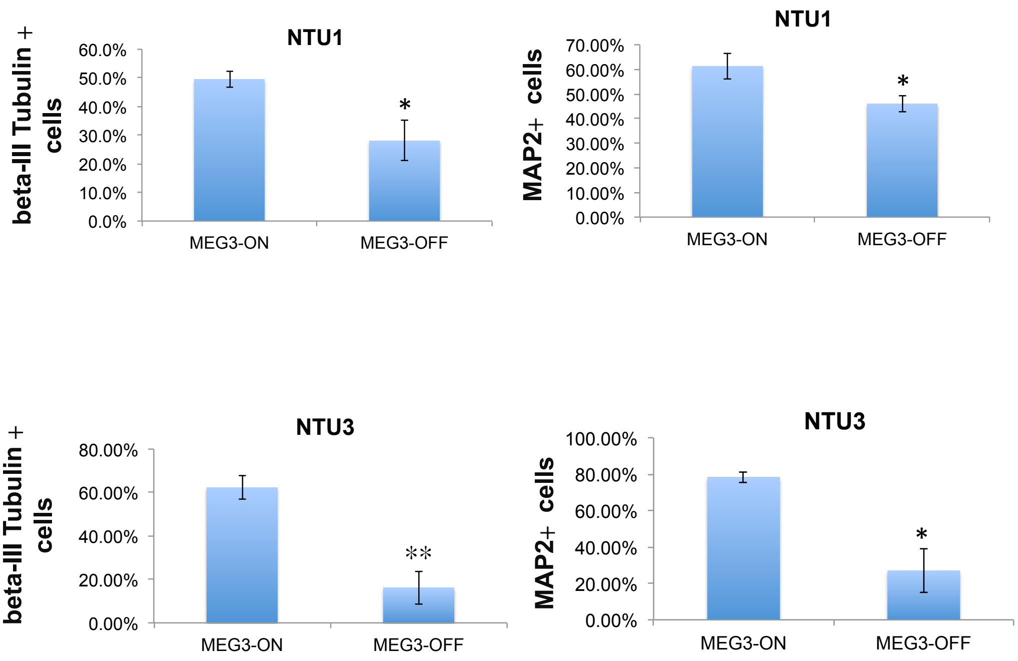

Figure S5. Quantitation of beta-III Tubulin or MAP2 positive cells after differentiation from the *MEG3*-ON and *MEG3*-OFF hESCs.

The percentage of beta-III Tubulin positive or MAP2 positive cells differentiated from the NTU1 and NTU3 *MEG3*-OFF hESCs was significantly lower than those from the *MEG3*-ON hESCs. Error bars represent the SEM generated from 2 biological samples with 3 technical repeats each. *P<0.05, ** P<0.01 compared with the corresponding *MEG3*-ON groups by Student's *t*-test.
