# Supplementary material for: Loss of non-coding RNA expression from the DLK1-DIO3 imprinted locus correlates with reduced neural differentiation potential in human embryonic stem cell lines
Source: Stem Cell Res Ther. 2015 Jan 5;6(1):1. doi: 10.1186/scrt535 (PMC4417332; doi:10.1186/scrt535)
Supplement: Supplementary file 8 — Additional file 8: Figure S6: Ki67 staining for the MEG3-ON and MEG3-OFF hESC-differentiated neural lineage-like cells after 18 days on Matirgel. The numbers of Ki67-positive-stained cells were not obviously different between the MEG3-ON and MEG3-OFF hESC-differentiated cells, suggesting that those observed differentiation defects seemed not to be linked to the change in cellular proliferation. Scale bars, 100 μm. hESC, human embryonic stem cell; MEG3, maternally expressed gene 3. (PDF 2 MB) [file 13287_2014_417_MOESM8_ESM.pdf]

Figure S6

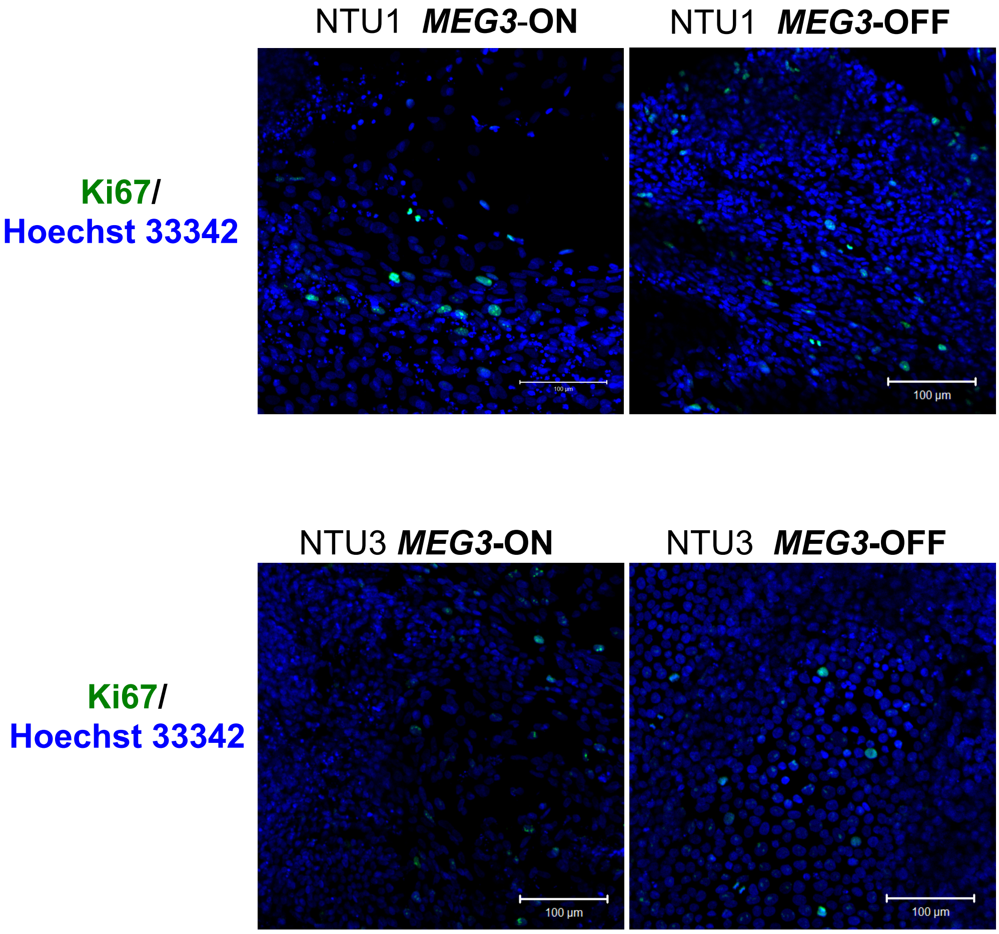

Figure S6. Ki67 staining for the *MEG3*-ON and *MEG3*-OFF hESC-differentiated neural lineage-like cells after 18 days on Matirgel.

The numbers of Ki67 positive-stained cells were not obviously different between the *MEG3*-ON and *MEG3*-OFF hESC-differentiated cells, suggesting those observed differentiation defects seemed not linked to the change in cellular proliferation. Scale bars, 100 µm.
